# Blood Biomarkers of Glioma in Response Assessment Including Pseudoprogression and Other Treatment Effects: A Systematic Review

**DOI:** 10.3389/fonc.2020.01191

**Published:** 2020-08-14

**Authors:** Istafa J. Raza, Campbell A. Tingate, Panagiota Gkolia, Lorena Romero, Jin W. Tee, Martin K. Hunn

**Affiliations:** ^1^Department of Neurosurgery, The Alfred Hospital, Melbourne, VIC, Australia; ^2^The Ian Potter Library, The Alfred Hospital, Melbourne, VIC, Australia; ^3^Department of Surgery, Faculty of Medicine, Nursing and Health Sciences, Monash University, Melbourne, VIC, Australia

**Keywords:** glioma, circulating biomarkers, exosomes, circulating nucleic acids, circulating tumor cells, treatment effects, pseudoprogression, response assessment

## Abstract

Imaging-based monitoring of disease burden in glioma patients is frequently confounded by treatment effects. Circulating biomarkers could theoretically augment imaging-based response monitoring. This systematic review aimed to present and evaluate evidence for differential expression and diagnostic accuracy of circulating biomarkers with respect to outcomes of tumor response, progression, stable disease, and treatment effects (pseudoprogression, radionecrosis, pseudoresponse, and pseudolesions) in patients undergoing treatment for World Health Organization grades II–IV diffuse astrocytic and oligodendroglial tumors. MEDLINE, EMBASE, Web Of Science, and SCOPUS databases were searched until August 18, 2019, for observational or diagnostic studies on multiple circulating biomarker types: extracellular vesicles, circulating nucleic acids, circulating tumor cells, circulating proteins, and metabolites, angiogenesis related cells, immune cells, and other cell lines. Methodological quality of included studies was assessed using an adapted Quality Assessment of Diagnostic Accuracy Studies-2 tool, and level of evidence (IA–IVD) for individual biomarkers was evaluated using an adapted framework from the National Comprehensive Cancer Network guidelines on evaluating tumor marker utility. Of 13,202 unique records, 58 studies met the inclusion criteria. One hundred thirty-three distinct biomarkers were identified in a total of 1,853 patients across various treatment modalities. Fifteen markers for response, progression, or stable disease and five markers for pseudoprogression or radionecrosis reached level IB. No biomarkers reached level IA. Only five studies contained data for diagnostic accuracy measures. Overall methodological quality of included studies was low. While extensive data on biomarker dysregulation in varying response categories were reported, no biomarkers ready for clinical application were identified. Further assay refinement and evaluation in larger cohorts with diagnostic accuracy study designs are required.

**PROSPERO Registration**: CRD42018110658.

## Background

### Treatment Effects in Glioma

Glioma is the most common primary brain tumor, making up 25.5% of all central nervous system (CNS) malignancies, with an incidence of 25,000 new cases per year in the United States (1) and pooled global incidence of 3.38 per 100,000 person-years (2). Survival outcomes for high-grade glioma (HGG) remain very poor.

Follow-up of glioma patients during and after standard treatment can be confounded by treatment-related effects (TEs) that can mimic tumor progression (TP). Pseudoprogression (PsP) is a TE that typically occurs 3–6 months after chemoradiotherapy (CRTx), although delayed manifestation is possible, with an incidence of up to 36% in HGG (3). It is characterized by new enhancement or T2/fluid-attenuated inversion recovery (FLAIR) signal that stabilizes or reduces without salvage therapy (4). Radionecrosis is a term commonly used interchangeably with PsP and is a frequent pathological feature (5). Pseudoprogression can be symptomatic because of edema and mass effect. Immune checkpoint inhibitors have also been reported in trials to induce increased lesion size or “pseudolesions” (PsLs) (6, 7). Conversely, antiangiogenic treatments, such as the anti–vascular endothelial growth factor (VEGF) monoclonal antibody bevacizumab, can induce marked reduction in enhancement or FLAIR signal through reduction of vasogenic edema, in a phenomenon termed “pseudoresponse” (PsR), without necessarily improving overall survival (OS) (5, 8, 9).

To address TEs, Response Assessment in Neuro-Oncology (RANO) criteria for HGG assert that a diagnosis of TP cannot be made in the first 12 weeks after CRTx completion unless new enhancement is outside the radiation field or via repeat tissue diagnosis, regardless of symptom status (5).

### Circulating Biomarkers in Glioma

**Figure 1:**
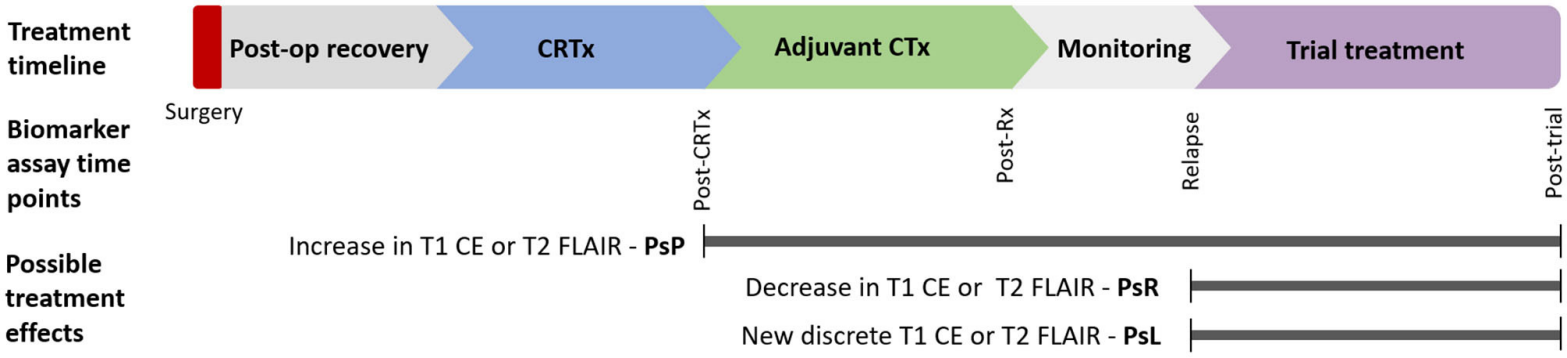
Postulated timepoints at which circulating biomarker could enhance monitoring of glioma therapy: Circulating biomarkers have several possible applications in glioma diagnosis and treatment across disease course. Various imaging findings across this timeline present distinct diagnostic uncertainty. This review focuses on biomarkers in response assessment during conventional and trial treatments. CE, contrast enhancement; CRTx, chemoradiotherapy; CTx, chemotherapy; FLAIR, fluid-attenuated inversion recovery; m, months; Post-op, postoperative; PsP, pseudoprogression; PsR, pseudoresponse; PsL, pseudolesions; Rx, treatment.

Circulating glioma biomarkers are a novel modality that could potentially augment response assessment. Scope exists for these to act as either triage tests or add-ons to standard imaging paradigms (Figure 1). There is also potential that circulating markers could be used in combination with novel imaging modalities such as magnetic resonance (MR) spectroscopy, positron emission tomography (PET), or MR perfusion to improve response assessment.

Several classes of circulating tumor biomarker have been reported (10–12). These can be broadly categorized into tumor-derived extracellular vesicle (EV) biomarkers, circulating cellular biomarkers, circulating nucleic acids (CNAs), and circulating protein markers. Notably, EVs and cellular markers may act as vehicles for genetic and protein markers. Extracellular vesicles can be subdivided into exosomes derived from multivesicular bodies, and microparticles (MPs) or microvesicles (MVs) derived directly from cellular plasma membrane. Extracellular vesicles are enriched in glioma-associated macromolecules, such as nucleic acids, mutant oncoproteins, and angiogenic proteins (13).

Cellular markers include circulating tumor cells (CTCs) (14), circulating endothelial cells (CECs), and circulating endothelial progenitor cells (CEPs) (15), as well as changes to other circulating cells of hematopoietic lineage including neutrophils, monocytes, natural killer (NK) cells, lymphocytes, and platelets. Circulating tumor cells result from epithelial-to-mesenchymal transition and subsequent invasion into the bloodstream (16). Circulating endothelial cells and CEPs are related to angiogenic cascade activation, with CECs thought to be derived from mature vessel turnover, whereas CEPs are bone marrow derived, fueling neoangogenesis, and are phenotypically similar to hematopoietic stem cells (15). Both CEC and CEP counts are elevated in glioma patients (17, 18).

Glioma CNAs include (i) circulating tumor DNA (ctDNA), a subset of normal cell-free DNA (cfDNA) complement derived from necrotic or apoptotic tumor cells; (ii) micro-RNAs (miRNAs), which are small non-coding RNAs 19–22 nucleotides in length that are found in blood either complexed with Argonaute effector proteins or in lesser proportion packaged within EVs (13, 19–22); and (iii) long non-coding RNAs (lncRNAs), which are 200-nucleotide-long RNA transcripts (23).

### Review Objectives

The primary objective of this systematic review is to identify and compare circulating biomarkers that are differentially expressed in different treatment response categories of TP, tumor response (TR), stable disease (SD), and TEs. The secondary objective of this review is to evaluate diagnostic accuracy of these biomarkers in differentiating response categories.

## Methods

### Literature Search Strategy and Selection Criteria

This systematic review was conducted according to PRISMA guidelines with preregistration of review protocol with the PROSPERO database in October 2018. To identify blood biomarkers, we searched literature databases MEDLINE, EMBASE, Web of Science, and Scopus to August 18, 2019, using MESH terms and keywords including “tumor progression,” “tumor recurrence,” “pseudoprogression,” “treatment effects,” “serum or blood or plasma markers,” “miRNA,” “ctDNA,” “lncRNA,” “extracellular vesicles,” “circulating tumor cells,” “circulating endothelial cells,” and “circulating endothelial progenitor cells” (detailed description of MEDLINE search in Table A1, Additional File 1).

We included any study on patients with histologically proven World Health Organization (WHO) grades II–IV diffuse astrocytic and oligodendroglial tumors that measured biomarker expression during or after adjuvant treatment and reported relevant outcomes as specified:

- For the primary objective, any measure of differential expression of biomarker at time of response categories of TR [including partial response (PR) and complete response (CR)], TP, SD, and any TEs including PsP, PsR, and PsL.
- For the secondary objective, any measure of the diagnostic accuracy of the biomarker in differentiating response categories including sensitivity (Sn), specificity (Sp), positive and negative likelihood ratios (+LR and –LR), diagnostic odds ratio (DOR), or area under the curve (AUC).

We did not place any limitation on reference standard for response classification, publication date, study design, or adjuvant treatment modality. When studies had overlapping cohorts, we included only the study with the largest cohort unless studies on smaller cohorts reported distinct biomarkers allowing inclusion for those biomarkers only. We excluded studies containing only WHO grade I glioma or those that included other CNS malignancies without analyzing outcomes in a glioma-only subgroup. Studies including WHO I glioma as part of a larger cohort with glioma of higher grades were included. We excluded studies where data were not extractable, pediatric populations, on cerebrospinal fluid (CSF), or other biofluid markers, or those unavailable in English. Studies published as abstracts were included if sufficient data were available for cohort size, histology, and outcome reporting. Outcomes defined by the histological finding of a pathological process other than tumor such as radiation necrosis or gliosis were treated as part of the clinical entity of PsP (5).

For inclusion, two authors (IR and PG) screened search results alongside references of relevant reviews with disagreements resolved by a third author (MH). Articles were excluded on title alone if it was clear that they were outside the inclusion criteria (e.g., review works, non-glioma, *in vitro*, animal, or pediatric studies). Abstracts were perused if deemed from the title that the study could theoretically include data related to the research question. Relevant full texts of articles were then perused unless the study could be excluded based on abstract alone.

### Data Extraction and Management

Data extraction was independently performed by two authors (IR and PG), with disagreements resolved by a third author (MH). Data were extracted into a standardized template, and included study characteristics were biomarker type, assay methodology, study design, size of glioma cohort in which biomarker was assayed, histology, treatment, relevant biomarker sampling timepoints, and reference standard. Any form of outcome data on differential expression in delineated response categories was reported. For studies that measured biomarkers at multiple timepoints, we extracted only those timepoints matched with reference standard. Diagnostic accuracy measures were calculated when not reported if adequate data were available. For studies including WHO I along with higher glioma grades that analyzed WHO II–IV glioma as separate subset(s), data on WHO I were excluded. Data on WHO I were included only if combined with cohorts including higher grades. Statistical measures were reported if available, taking *p* < 0.05 as cutoff for significance.

#### Assessment of Methodological Quality

Two authors (IR and CT) independently appraised the methodological quality of included studies using an adapted Quality Assessment of Diagnostic Accuracy Studies-2 (QUADAS-2) tool (24) (Table A2, Additional File 1), with disagreements resolved by a third author (PG). For abstracts, study authors were contacted for further information to allow quality assessment. If authors did not respond, quality assessment of abstracts was not performed.

#### Data Synthesis

Overall level of evidence (LOE) for individual biomarkers was appraised using an adapted framework in accordance with the National Comprehensive Cancer Network (NCCN) Task Force: Evaluating the Clinical utility of Tumor Markers in Oncology guidelines (25) (levels IA–IVD, Table A3, Additional File 1). Briefly, the index study for each biomarker was the positive study with the highest level study design. Other studies were used to evaluate consistency in data. Abstracts without adequate information to apply this framework were excluded from LOE analysis. Biomarkers with no positive outcome reported were rated as not applicable (NA).

Meta-analysis was not performed because of anticipated heterogeneity of included markers and outcomes. Reporting was stratified by biomarker subtype and treatment at time of biomarker sampling. Biomarker subtypes were divided into EV-derived, CNA, CTC, angiogenesis, and inflammation related proteins, circulating angiogenesis-related cells, immune and other circulating cell types, and other circulating proteins. Treatment subgroups were standard therapy [cytoreductive chemotherapy (CTx) +/– radiotherapy (RTx)], immunotherapy, antiangiogenic therapy, or combined therapy/other modalities/not specified.

## Results

**Figure 2:**
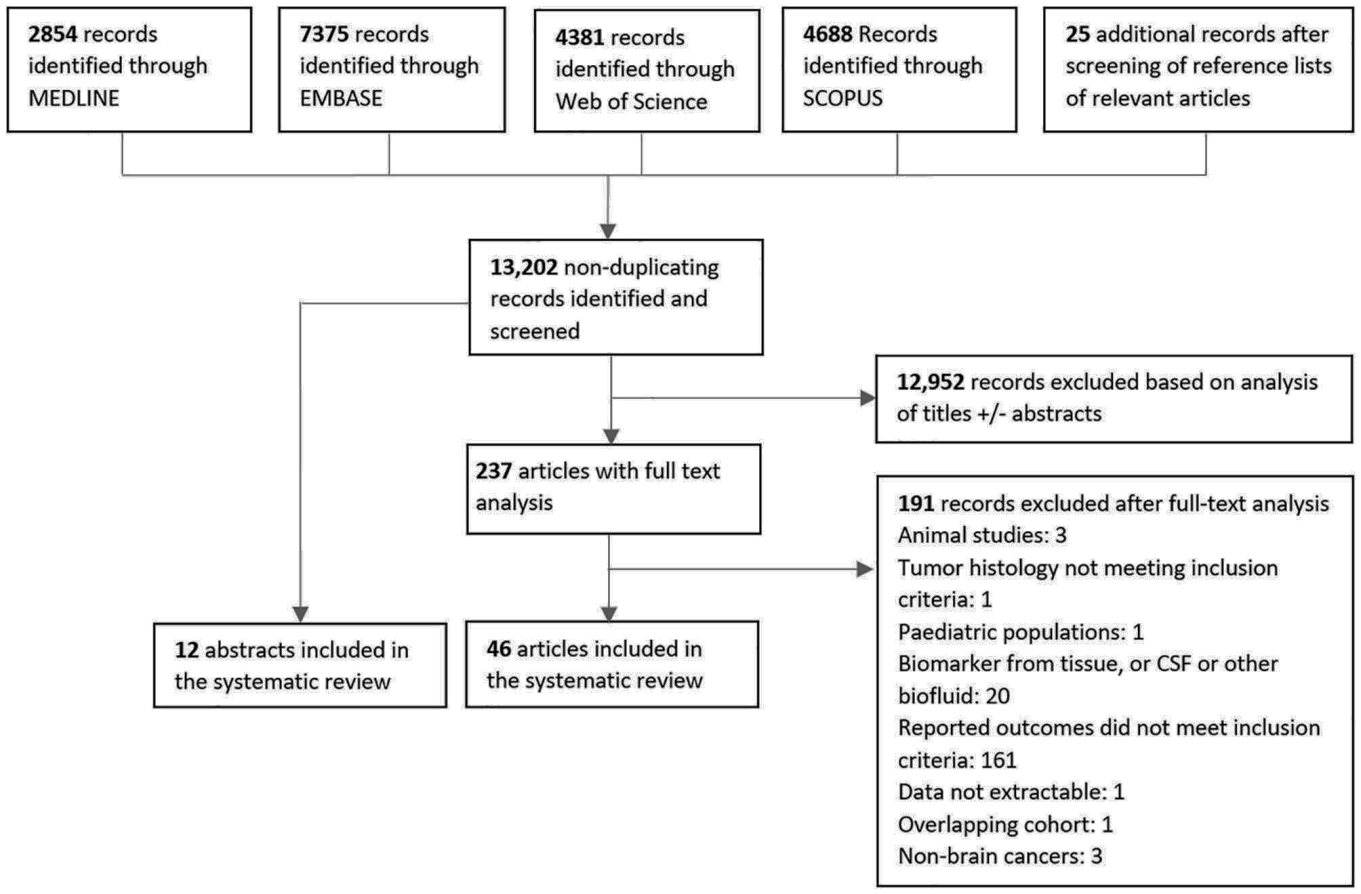
PRISMA diagram for screening, exclusion and inclusion of studies in this systematic review.

After screening a total of 13,202 non-duplicating published records, a total of 58 studies met the inclusion criteria consisting of 12 abstracts and 46 articles (Figure 2). The vast majority of *in vitro*, animal, and pediatric studies were excluded prior to reaching full-text analysis. The major reasons for exclusion of full-text articles were if data on response related outcomes were not present (161 articles), or if biomarkers were in CSF or another biofluid (20 articles). One hundred thirty-three distinct biomarkers were identified and studied in a total of 1,853 patients with a mean sample size of 31 patients per study (range = 1–343). Twenty-nine studies reported biomarker outcomes during standard treatment, eight studies during antiangiogenic therapy, four during immunotherapy modalities, and 20 under combined therapies/other/not specified. Characteristics of included studies are summarized in Tables A4–10 (Additional File 2).

### Tumor Marker Subtypes

#### Extracellular Vesicle–Derived Biomarkers

Five studies reporting 12 separate EV-derived biomarkers were identified (Table A4) (26–30). Alterations in EV count and expression profile at time of TP or TR were established in four studies. Total EV concentration was higher in glioblastoma (GBM) TP cases vs. postoperative cases (IB) (26). Similarly, MPs expressing glial fibrillary acidic protein (GFAP) and the procoagulant tissue factor were shown to rise during follow-up in TP but not SD cases after standard RTx + temozolomide (IIB) (27). Separately, using microfluidic chip based immunomagnetic exosomal isolation coupled with micro-nucleic magnetic resonance spectroscopy (micro-NMR), Shao et al. (30) demonstrated higher “drug efficacy index” among TR cases compared to those without response to treatment with RTx + temozolomide +/– trial therapy in a prospective controlled trial (PCT) (IB). This index was calculated based on decreases in MV counts and reduction in MV expression of prototypical GBM oncoproteins epidermal growth factor receptor (EGFR), EGFRvIII, PDPN, and IDH1-R132H. In an example of therapy-specific monitoring, survivin-expressing exosomes were elevated in early but not late TP cases after trial vaccination with survivin epitope (IIB) (28). Only one study evaluated for differential expression in TP vs. PsP. Annexin V^+^/EGFR^+^ MV counts were elevated in TP vs. PsP cases across multiple sampling timepoints matched to MR imaging (MRI) scans assessed via RANO criteria in a prospective cohort of 11 GBM patients (IB) (29).

#### Circulating Nucleic Acids

Twelve studies reporting 19 different CNA biomarkers were identified (Table A5) (31–42). Among these, 14 were miRNAs. All were assayed using quantitative reverse transcription polymerase chain reaction (qRT-PCR). Two studies used an exosomal source comprising four miRNAs (32, 33). In three studies, miRNAs reached level IB evidence for association between altered circulating quantity and worsened disease status. miR-301a and miR-205 were elevated in GBM cases at time of TP vs. postoperatively in two separate longitudinal cohorts (32, 38). While the strength of these two studies was their prospective design, reference standard was not reported. In a separate study, miR-221 and miR-222 displayed sequentially elevated expression in worsening response categories CR, PR, SD, and TP (36). Two additional miRNAs (miR-21 and miR-124-3p) reached LOE IVD in case reports showing elevation prior to TP. Santangelo et al. reported a cumulative rise in a panel of three miRNAs; miR-21, miR-124-3p, and miR-222 2 months prior to confirmation of a case of GBM TP (33). Ilhan Mutlu et al. (31) also reported a single GBM case of miR-21 rise prior to TP. In notably conflicting findings, a study by Siegal et al. (35) reporting no correlation between change in miR-21 and miR-10b with response outcome as assessed by the RANO criteria in separate HGG cohorts treated with either temozolomide or bevacizumab.

For differential expression in PsP specifically, the highest LOE was IVD, incorporating seven miRNAs. A second case in the report by Santangelo et al. (33) showed cumulative miRNA decrease prior to PsP confirmation. Separately, Yang et al. (37) reported that 4 miRNAs initially discovered via high-throughput Solexa sequencing and validated with qRT-PCR, miR-150,−197,−23a, and−548-5p, were all elevated in PsP cases, when compared to WHO II, III, and IV glioma cohorts in preoperative blood samples. Diagnostic performance of the cumulative miRNA panel was high with AUC = 0.950 [95% confidence interval (CI), 0.902–0.998], but the main study limitation was cross-sectional design (37).

Four studies on circulating DNA were identified. Using a commercial next-generation sequencing platform, ctDNA mutation frequency was higher in cases demonstrating TP within <30 vs. >30 days of assay, with recruitment from an observational registry (IIIC) (39). Calculated diagnostic performance for TP <30 days was low, with Sn = 56%, Sp = 79%, +LR = 2.70, –LR = 0.55, DOR = 4.91 (39). Cordova et al. (42) reported a case series on IDH1 wild-type GBM in which presence and levels of C228T/C250T mutated TERT ctDNA, a mutation commonly demonstrated in diffuse glioma tissues, correlated with SD, TP, and PsP outcomes in selected patients (IVD). Total cfDNA, measured using fluorometric methods, was found to correlate with disease trajectory (TP or PsP) in two case series (IVD) (40, 41).

A single study on exosomal mRNA was identified, in which the Shao group demonstrated, using microfluidic qRT-PCR, that change in exosomal mRNA levels for DNA repair genes MGMT and APNG showed association with response status of TR, SD, or TP during treatment with temozolomide in case series (IVD) (34). No studies meeting inclusion criteria for lncRNAs were identified.

#### Circulating Tumor Cells

Four studies met the inclusion criteria on glioma CTCs (Table A6) (43–46). Use of a microfluidic device with antibodies targeted at cancer markers SOX2, tubulin β3, EGFR, A2B5, and c-MET (“STEAM” cocktail) detected CTC in 39% of GBM cases (46). In this cohort, the median CTC count in postoperative TP samples was higher than in those with SD (IIC) (46). Three reports using either serial culture, human telomerase probe, or polyploid chromosome 8 FISH also observed CTC rise with TP in HGG (43–45). Moreover, post-treatment CTC reduction was also demonstrated in PsP cases (IVD) (43, 44). Overall, CTC studies for response monitoring in HGG were limited by assay sensitivity and low sample size.

#### Angiogenic and Inflammatory Signaling Molecules

Forty-nine biomarkers within 15 studies were proteins related to angiogenic and inflammatory signaling cascades (Table A7) (47–61). The vast majority were detected using enzyme-linked immunosorbent assay (ELISA) or other immunoassay variants. Ten of 15 studies reporting biomarkers in this category involved treatment with an antiangiogenic agent either alone or in combination with other agents (52–57, 59–61). Antiangiogenic agents used in identified studies were bevacizumab, cediranib [a VEGF receptor (VEGFR) inhibitor], aflibercept [a VEGF and placental growth factor (PIGF) decoy receptor], vorinostat (a histone deacetylase inhibitor with antiangiogenic properties) and vandetanib (a VEGFR inhibitor). Six of 15 studies were PCTs on trial treatment with biomarkers as secondary outcomes (48, 51–53, 56, 59), and a further two studies relied on ancillary recruitment from trials (47, 55). Nine of 15 were on recurrent HGG populations (51–58, 61). The highest LOE biomarker was angiopoietin-2 (Ang2) (a proangiogenic growth factor) (IB), which showed increase at TP vs. two and four cycle timepoints during bevacizumab treatment in PCT (54). However, a similar finding for Ang2 was not observed in cediranib or vandetanib trial (52, 59).

At LOE IIB, biomarkers included the matrix metalloproteinases MMP2 and MMP9 and their endogenous inhibitor TIMP1. Notably, MMP9 was the subject of the largest cohort study in this review by Iwamoto et al. (47), consisting of 343 glioma patients, which found no association between MMP9 levels and disease status in either low-grade glioma (LGG) or HGG. Likewise, MMP9 was not dysregulated at TP during bevacizumab + irinotecan for recurred HGG (61). However, MMP9 was found to be elevated with worsened response during aflibercept trial (53). Other biomarkers related to angiogenic signaling at IIB included proangiogenic growth factors VEGF-A, basic fibroblast growth factor, platelet-derived growth factor, and PIGF, and circulating receptors sVEGFR1 and sTie2 (a serum angiopoietin receptor). Vascular endothelial growth factor-A is notable as it was the most frequently studied biomarker in this category, in a total of eight studies, with conflicting results. Association between TP and elevation in VEGF-A was reported during bevacizumab + irinotecan (57), as well as during intranasal monopterene perillyl alcohol trial therapy (58); however, the finding was not reflected in multiple other trials including two separate bevacizumab studies (54, 55), three other antiangiogenic agent trials (53, 56, 59), and one trial on cytoreductive temozolomide + thalidomide + celecoxib (48). Additional biomarkers at LOE IIB were the chemokines interleukin 8 and stromal cell derived factor 1α.

Two studies evaluated biomarkers from this class in PsP. During vantedanib trial, multiple biomarkers were shown not to be correlated with treatment response (ranking from better to worse CR, PR, SD, PsP, and TP) at timepoints matched with MRI follow-up (59). Interestingly, very early changes (within 4 h to 2 days) in three biomarkers (CA9, collagen IV, and sVEGFR2) were associated with eventual disease outcome; however, these were excluded from this analysis given the inappropriately long time interval between such changes and response assessment. A second study evaluating MMP2 and the inflammatory response protein NGAL did not find differential expression in PsP vs. TP (60).

#### Circulating Angiogenesis Related Cells

Five studies reported on four separate circulating cell populations hypothesized to be involved in glioma angiogenesis and vessel turnover, assayed using FACS utilizing specific surface markers (Table A8) (52, 62–65). Cuppini et al. (62) reported changes in counts of five separate cell populations; three CEC subtypes (CECs overall, viable CECs, CD109^+^ CECs), hematopoietic progenitor cells, and progenitor perivascular cells (IB). Notably, alterations in counts differed across response outcomes, as well as treatment modality. Bevacizumab + irinotecan induced reduction in counts of all colonies at 8 weeks posttreatment in those with either PR or SD but not TP. In those treated with bevacizumab alone, only the CD109^+^ CEC population showed reduction in PR/SD cases but not TP. No changes were seen in a cohort treated with conventional cytoreductive temozolomide/fotemustine. To add to evidence for CEC correlation with response outcome during antiangiogenic modality, viable CEC counts were shown to rise concurrent with TP during cediranib PCT (64) and were elevated in TP vs. TR/SD at trial end for combined sorafenib + bevacizumab (65). Less evidence was found for CPC (IVD), whereby only one case of CPC rise at TP was reported (63). Additionally, no CPC variation was found at time of TP during cediranib treatment (52). No studies reported relationship of circulating angiogenesis-related cells and TEs.

#### Alterations to Immune Related and Other Circulating Cell Populations

Thirty-seven biomarkers related to change in circulating immune cell and other hematopoietic lineage cells were reported in 14 studies (Table A9) (51, 55, 66–77). Six of these studies included treatment with dendritic cell (DC) vaccine immunotherapy with or without additional modalities (51, 71–73, 75, 77). Three studies involved bevacizumab treatment (55, 73, 74). Highest level of evidence (IIB) came from a PCT by Pellegatta et al. (73), where DC vaccine was used alongside conventional RTx/temozolomide in 24 patients with newly diagnosed GBM. Counts of CD8^+^ T cells, CD4^+^ T cells, and NK cells, as well as percentages of these cells expressing immune cell activation markers GZMB, ABCC3, and/or interferon γ (IFN-γ) were assayed. Selected subsets demonstrated elevation in cases maintaining TR/SD until 12-months follow-up without significant change in TP cases.

Case-level data were also found for higher frequency of immune responses in TR and SD vs. TP during DC vaccine (IVD). In a DC vaccine PCT, IFN-γ response to epitope stimulation in peripheral mononuclear cells detected via ELISpot assay was more common at time of either TR or SD vs. time of TP (72). A separate DC vaccine did not show such a trend for IFN-γ ELISpot; however, tetramer assay for antigen-specific CD8^+^ T cells did reveal more common responses in TR or SD vs. TP at 9 weeks (77). In a study of a WT1 epitope-loaded DC vaccine, tetramer assay-verified WT1-specific CD8^+^ T-cell responses were seen in 2 SD patients (75). During DC vaccine for recurrent GBM, TP was more common in those without epitope-specific cytotoxic CD8^+^ T-cell responses, with diagnostic performance for lack of response as a tool to diagnose TP calculated as Sn = 83%, Sp = 55%, +LR = 1.88, –LR = 0.30, DOR = 6.27 (71).

Among studies on immunological markers, there was a comparatively high proportion of studies on utility for differentiating PsP from TP (5 of 14) (66–70). Four biomarkers in two studies reached LOE IB. In operative biopsy confirmed GBM vs. PsP samples, the preoperative percentage of monocytic myeloid-derived suppressor cells (Mo-MDSCs) with negative/low expression of MHCII class molecule HLA-DR (Mo-MDSC HLA-DR^neg/low^) was elevated, whereas the percentage expressing inflammation regulator VNN2 was reduced (69). The ratio of HLA-DR^neg/low^ to VNN2 Mo-MDSCs (termed the DR-Vanin Index) was therefore higher in recurrent GBM vs. PsP cases. Three further LOE IB markers demonstrated by Parsa et al. were lower counts of activated NK2GD^+^ NK cells and NK2GD^+^ cytotoxic CD8^+^ T cells and higher counts of activated CD25^+^FOXP3^+^ regulatory T cells in TP cases vs. PsP cases (68). Differential neutrophil-lymphocyte ratio (NLR) was also identified in PsP vs. TP cases (LOE IIIC), with reduction of NLR associated with PsP cases post-RTx/temozolomide for GBM (67). Diagnostic performance for NLR decrease to detect PsP was low, calculated at Sn = 59%, Sp = 67%, +LR = 1.85, –LR = 0.60, and DOR = 3.08 (67).

#### Other Circulating Proteins

Eleven additional circulating proteins were identified in eight separate studies (Table A10) (55, 76, 78–83). One biomarker, YKL-40 glycoprotein, which has been implicated in glioma invasiveness (84), reached LOE IB. YKL-40 levels were lower in CR (disease absent) vs. PR/SD/TP cases (disease presence) for GBM and AA but not LGG in the Iwamoto group (78) cohort of 343 glioma cases. Diagnostic performance was, however low, with AUC (95% CI) of 0.65 (0.60–0.69) for anaplastic glioma and 0.65 (0.61–0.70) for GBM (78). Glial fibrillary acidic protein and a neural lineage marker NfL demonstrated elevation in TP vs. SD cases in a cross-sectional study (81); however, in a longitudinal study, only one of 18 cases showed GFAP elevation at TP, with GFAP remaining undetectable in the remainder (LOE IVD) (83). Serum autoantibodies to MGMT have also been reported in glioma, with increased epitope coverage and titer in TP cases (LOE IVD). A case series found no difference in expression of GFAP and other neural markers neurogranin, intercellular adhesion molecule-5, brain-derived neurotrophic factor, and B-synuclein between recurrent GBM and PsP cases (80).

### Quality Assessment of Individual Studies

**Figure 3:**
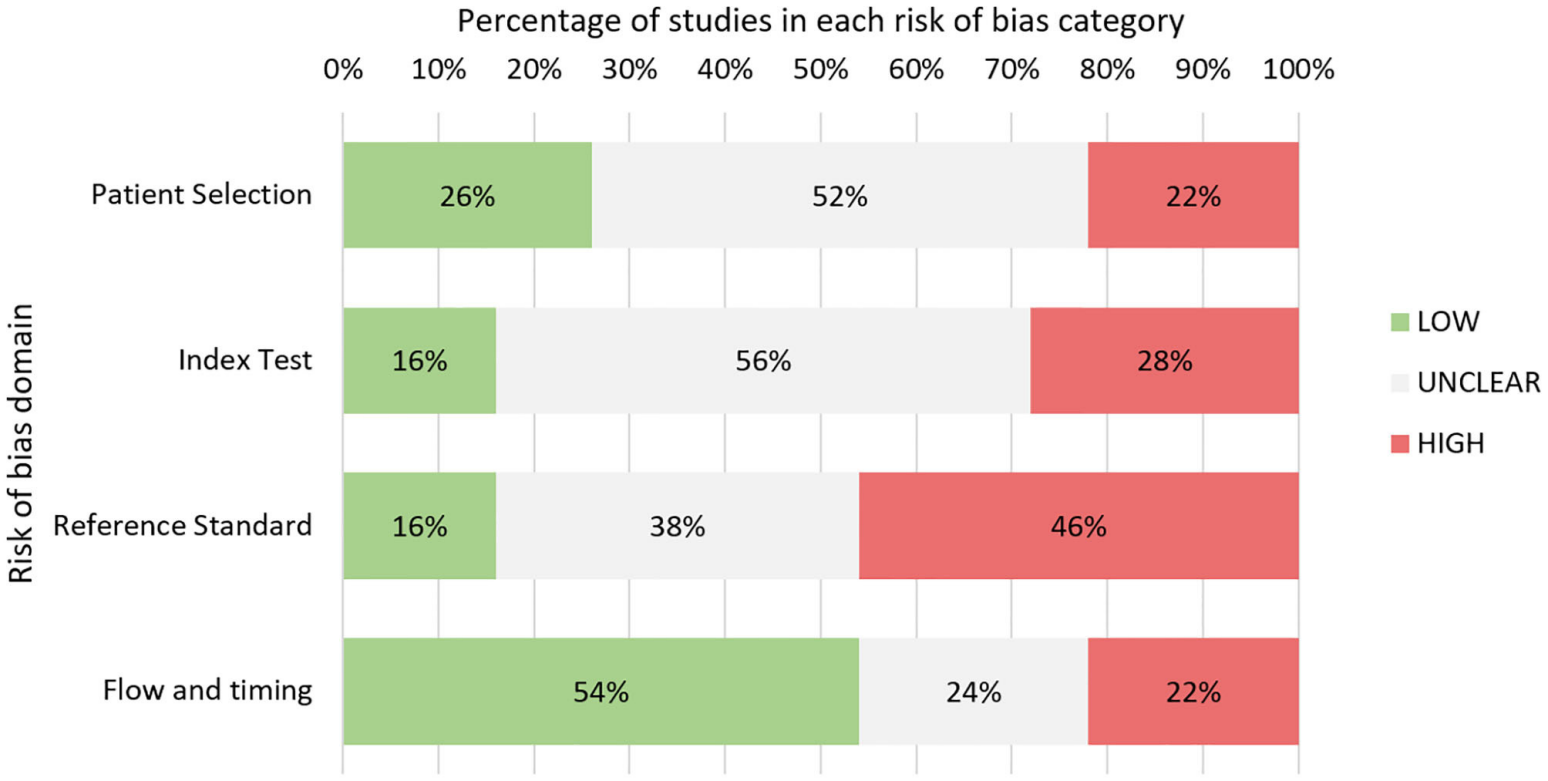
Quality assessment based on adapted QUADAS-2 tool: summary of findings.

Quality assessment was performed for 50 studies in total (Figure 3 and Table A11, Additional File 3). Eight abstracts were excluded as authors did not respond with adequate information. Overall, studies predominantly had either high or unclear risk of bias for three of four QUADAS-2 bias domains: patient selection, index test, and reference standard.

Notably, a high proportion of studies were either case reports or case series (15 in total), leading to high concerns for selection bias. In many studies, consecutive sampling was not specified leading to adjudgment of unclear risk of bias. In the index test domain, many studies lacked specific mention of blinding leading to unclear risk. A wide range of reference standards for response classification were used in these studies including imaging, histopathology, and multidisciplinary team consensus (Table 1). A large proportion of studies, 46%, were judged to have high concerns for bias in this domain. Twelve studies relied on the McDonald MRI criteria, which, unlike the latest RANO criteria, do not explicitly specify methods for exclusion of TEs (87). The RANO criteria were considered a robust classification standard. Seven studies involved histopathology as reference standard, which was considered low risk. Five studies utilized novel imaging techniques such as MR spectroscopy or PET as adjuncts to other tools. Many other studies failed to specify response assessment criteria leading to unclear risk.

**Table 1:** Summary of response assessment reference standards used for studies in this systematic review.

**Reference standard**	No. of studies^*^	**Strengths**	**Limitations**	**Likely to correctly classify**
MRI—RANO criteria	13	Clear guidelines on differentiating PsP and PsR from other response outcomes Specify criteria for diagnosing TP on histology when imaging is equivocal Most up-to-date consensus on glioma response assessment	Retrospective conclusion about final response outcome in equivocal cases can lead to diagnostic delay No criteria for PsLs	Yes
MRI—Macdonald criteria	12	Specifically designed for glioma Allows for inclusion and comparison of patients evaluated prior to introduction of RANO	Superseded by RANO In original form, no specific guidelines on excluding TEs Lack assessment of non-enhancing component of tumor Lack of guidance on multifocal lesions	No
MRI—RECIST criteria	2	Nil for glioma	Not specifically designed for glioma No specific guidelines on excluding TEs Superseded by glioma specific criteria	No
MRI—WHO criteria	1	Nil for glioma	Not specifically designed for glioma No specific guidelines on excluding TEs Superseded by RECIST criteria	No
MRI—criteria NOS	13	N/A	N/A	Unclear
Histopathology	7	Direct exclusion of confounding pathological processes When tissue sample adequate can act as gold standard for definitive diagnosis Risk of surgical and perioperative complications	Standardized guidelines may not be used for cases in which diagnosis is uncertain Only has utility in patients undergoing reoperation at TP Limitations in tissue sampling may not account for within-lesion heterogeneity	Yes
MR spectroscopy	4	Potential for time of test diagnosis Non-invasive Useful add-on test in equivocal cases	No standardized parameters for interpretation Only moderate diagnostic performance in TP vs. PsP (85)	Variable
PET imaging	1	Potential for time of test diagnosis Non-invasive Useful add-on tests in equivocal cases	No standardized parameters for interpretation Only moderate diagnostic performance in TP vs. PsP (86)	Variable
Multidisciplinary team consensus	2	Can use multiple data sources Reflects “real-world” diagnostic decisions	Not standardized, significant between team variation likely Blinding harder to maintain	Yes
Not specified	5	N/A	N/A	Unclear

* *Only studies included in quality assessment are counted. Some studies used more than one reference standard, either together or with variable reference standards for differing cases, in which case they are counted twice. Note that blinding was also taken into account for final risk of bias ratings for reference standard domain for individual studies, see Table A11*.

In the flow and timing domain, a larger proportion of studies were judged to have low risk of bias (54%), which was due to widespread use of predefined imaging response assessment criteria and appropriate biomarker sampling timepoints. This was partly because several studies were clinical trials on novel glioma therapies, which built blood sampling concurrent with standard MRI follow-up into the study design. An inappropriately long interval between index test and reference standard, defined as 1 month or greater, was implicated in eight of 11 studies adjudged to have high risk of bias in this domain, with time interval ranging from 1 to 4 months between biomarker sample and MRI. The remaining three studies with high risk of bias involved use of differing reference standards between patients.

### Summary of Findings and Levels of Evidence of Circulating Biomarkers

A total of 113 biomarkers within 46 studies had outcomes reported on differential expression in standard response categories of TP, TR, or SD (Table 2). Among these, 37 biomarkers were positive for differential expression in at least one response category, 28 had conflicting results and 48 were not found to have any differential expression in any response category. A total of 38 biomarkers within 14 studies had outcomes reported on differentiation of TP vs. PsP (Table 2). Among these, 19 biomarkers were positive (differential expression in TP vs. PsP), and 19 were negative. No studies reported biomarkers for PsL or PsR.

**Table 2:** Results and level of evidence ratings for biomarkers with differential expression in tumor progression, stable disease, and/or tumor response.

**Biomarker subtype**	**LOE**	**Biomarker**	**Standard treatment**	**Combination therapy/others**	**Immunotherapy**	**Anti-angiogenic therapy**
Extracellular vesicle biomarkers	IB	EV count	(+): (26, 27)	(+): (30)		
		MV count^i^		(+): (30)		
		MV EGFR^i^		(+): (30)		
		MV EGFRVIII^i^		(+): (30)		
		MV PDPN^i^		(+): (30)		
		MV IDH1-R132H^i^		(+): (30)		
	IIB	Exosome count			(+): (28)	
		GFAP+ EVs	(+): (27)		(–): (28)	
		MP counts	(+): (27)			
		SVN+ EVs			(+): (28)	
		TF+ EVs	(+): (27)			
Circulating nucleic acids	IB	miR-205	(+): (38)			
		miR-221	(+): (36)			
		miR-222	(+): (33, 36)			
		miR-301a	(+): (32)			
	IIIC	ctDNA (mutation frequency)		(+): (39)		
	IVD	APNG mRNA	(+): (34)			
		cfDNA (total)		(+): (40, 41)		
		MGMT mRNA	(+): (34)			
		miR-124-3p	(+): (33)			
		miR-21	(–): (35) (+): (31, 33)			(–): (35)
	NA	miR-10b	(–): (35)			(–): (35)
Circulating tumor cells	IIC	CTC	(+): (43–46)			
Angiogenesis and inflammation	IB	Ang2		(–): (59)		(–): (52) (+): (54)
related signaling molecules	IIB	bFGF	(–): (48)	(–): (57, 59)		(–): (53) (+): (52)
		IL-8		(–): (57) (+): (58)^*^		(–): (53) (+): (52)
		MMP2		(+): (61)		(–): (52)
		MMP9	(–): (47)	(–): (61)		(+): (24)
		PDGF		(–): (57) (+): (56), (58)^*^		
		PIGF		(–): (59)		(–): (53, 54)(+): (52)
		SDF1α		(–): (59)		(–): (53)(+): (52)
		sTie2		(–): (59)		(–): (54)(+): (52)
		sVEGFR1		(–): (59)		(+): (52)
		TIMP1	(–): (50)			(+): (53)
		VEGF-A	(–): (48)	(–): (55, 56, 59) (+): (57, 58)^*^		(–): (53, 54)
	IIIC	FVIII				(+): (55)
		IGFBP-2	(+): (49)	(–): (56)		
		vWF				(+): (55)
	NA	Ang1				(–): (52)
		Angiogenin		(–): (56)		
		Angiostatin		(–): (56)		
		CA9		(–): (59)		(–): (53)
		Collagen IV		(–): (59)		
		D-Dimer				(–): (55)
		Endostatin	(–): (48)			
		F1+2		(–): (55)		
		G-CSF		(–): (57)		
		IGF-1		(–): (56)		
		IGF-1 sR		(–): (56)		
		IGF-2		(–): (56)		
		IGFBP-1		(–): (56)		
		IGFBP-3		(–): (56)		
		IGFBP-4		(–): (56)		
		IGFBP-5		(–): (56)		
		IGFBP-6		(–): (56)		
		IL-1β				(–): (52)
		IL-6				(–): (52, 53)
		IL-12		(–): (57) (–): (51)		
		IL-13		(–): (57)		
		IL-17		(–): (57)		
		MIP-1β		(–): (57)		(–): (53)
		MMP10				(–): (52)
		PAI-1		(–): (55)		
		SCGFβ				(–): (53)
		sVEGFR2		(–): (56) (–): (59)		(–): (53)
		sVEGFR3		(–): (56)		
		TAT		(–): (55)		
		TGFα				(–): (52)
		TSP-1	(–): (48)			
		VEGF-C		(–): (56)		
		VEGF-D		(–): (56)		
Angiogenesis related circulating cells	IB	CEC (CD 109+/viable CEC/overall CEC)	(–): (62)	(+): (62)		(+): (62, 64, 65)
		HPC	(–): (62)	(+): (62)		(–): (62)
		PPC	(–): (62)	(+): (62)		(–): (62)
	IVD	CPC	(–): (62)	(–): (62) (+): (63)		(–): (52, 62, 64)
Alterations to immune related and other circulating cell populations	IIB	NK cells ABCC3+		(+): (73)		
		NK cells CD56 dim		(+): (73)		
		NK cells GZMB+		(+): (73)		
		NK cells IFN-γ+		(+): (73)		
		Lymphocytes		(–): (55) (+): (73)		
		PBL ABCC3		(+): (73)		
		PBL IFN-γ		(–): (51) (+): (73)		
		T-cells CD4+ ABCC3+		(+): (73)		
		T-cell CD4+ IFN-γ+		(+): (73)		
		T-cell CD4+		(+): (73)		
		T-cell CD8+		(–): (51) (+): (73, 75)		
	IVD	Monocytes		(+): (74)		
		Mononuclear cells IFN-γ		(–): (77) (+): (72)		
		Neutrophils		(–): (55) (+): (74, 76)		
		T-cell CD4+:CD8+ ratio		(+): (74)		
		T-cells CD8+ antigen specific		(+): (75, 77)	(+): (71)^*^	
		PBL E4BP4		(+): (51)		
		Platelets		(–): (55) (+): (76)		
		WCC		(–): (74) (+): (76)		
	NA	B-cells		(–): (74)		
		Dendritic cells		(–): (74)		
		Eosinophils		(–): (74)		
		Leukocytes		(–): (74)		
		MPV		(–): (55)		
		NK cells	(–): (70)	(–): (51, 73, 74)		
		NK cells CD56 bright		(–): (73)		
		PBL granzyme B		(–): (51)		
		PDW		(–): (55)		
		T-cells CD8+ ABCC3+		(–): (73)		
		T-cells CD8+ GZMB+		(–): (73)		
		T-cells CD8+ IFN-γ+		(–): (73)		
Other protein	IB	YKL-40	(+): (78)			
biomarkers	IVD	GFAP		(–): (83) (+): (81)		
		MGMT Autoantibodies	(+):(79)			
		Hemoglobin		(–): (55) (+): (76)		
		NfL		(+): (81)		
	NA	Recoverin		(–): (82)		
		Tau protein		(–): (81)		

*(+), positive finding; (–), negative finding; ABCC3, ATP binding cassette subfamily C member 3; Ang, angiopoeitin; APNG, alkylpurine DNA n-glycosylase; bFGF, basic fibroblast growth factor; CA9, carbonic anhydrase 9; CD, cluster of differentiation; CEC, circulating endothelial cell; cfDNA, cell-free DNA; CTC, circulating tumor cells; CPC, circulating progenitor cell; ctDNA, circulating tumor DNA; EGFR, epidermal growth factor receptor; EGFRvIII, EGFR variant III mutation; EV, extracellular vesicles; F1+2, prothrombin fragment 1+2; FVIII, factor VIII; G-CSF, granuocyte colony stimulating factor; GFAP, glial fibrillary acidic protein; GZMB, granzyme b; HPC, haematopoetic progenitor cells; IDH-R132H, cytosolic isocitrate dehydrogenase 1 mutation; IGF, insulin like growth factor; IGFPBP, insulin like growth factor binding protein; IL, interleukin; IFN, interferon; LOE, level of evidence rating; MGMT, O-6-methyl-guanine-DNA-methyl-transferase; MIP-1β, macrophage inflammatory protein-1β; miR, microRNA; MMP, matrix metalloproteinase; MP, microparticle; MPV, mean platelet volume; mRNA, messenger RNA; MV, microvesicle; NfL, neurofilament light chain; NGAL, neutrophil gelatinase-associated lipocalin; NK, natural killer; PAI-1, plasminogen activator inhibitor-1; PBL, peripheral blood lymphocytes; PDGF, platelet derived growth factor; PDPN, podoplanin; PDW, platelet distribution width; PIGF, placental growth factor; PPC, progenitor perivascular cell; SCGFβ, serum stem cell growth factor β; SDF1α, stromal cell derived factor 1α; sTie2, soluble Tie-2; sVEGFR, soluble VEGF receptor; SVN, survivin; TAT, thrombin-antithrombin complexes; TF, tissue factor; TGF- α, transforming growth factor α; TIMP1, tissue inhibitor of matrix metalloproteinase 1; TSP-1, thrombospondin 1; VEGF, vascular endothelial growth factor; vWF, von Willebrand Factor; WCC, white cell count; YKL-40, chitinase 3-like 1 glycoprotein*.

* *Abstract for ref. (58) included in LOE analysis as insufficient information available*.

i *These biomarker were incorporated into a drug efficacy index, see Table A4 for further details*.

Only two studies reported diagnostic performance measures—one study for differentiation of CR (disease absence) vs. PR/SD/TP (disease presence) (YKL-40) and one study for differentiation of RN vs. active disease (miR-23a, miR-150, miR-197, and miR-548-5p). A further three studies reported data enabling calculation of diagnostic performance measures—two on TP (ctDNA mutational frequency and T-cell response during immunotherapy) and one on TP vs. PsP (reduction in NLR).

Level of evidence ratings were performed for 133 biomarkers from 56 studies in total. Two abstracts were excluded from LOE analysis as inadequate information was available. For differential biomarker expression in TR, SD, or TP (Table 3), no biomarkers reached level IA, 15 reached IB, 27 reached IIB, 1 reached IIC, 4 reached IIIC, 18 reached IVD, and 49 were not rated (NA). For differential expression in TP vs. PsP (Table 2), no biomarkers reached IA, 5 reached IB, none reached IIB, none reached IIIC, 10 reached IVD, and 23 were not rated (NA).

**Table 3:** Results and levels of evidence ratings for biomarkers with differential expression in TP vs PsP.

**Biomarker subtype**	**LOE**	**Biomarker**	**Standard treatment**	**Combination therapy/others**	**Immunotherapy**	**Anti-angiogenic therapy**
Extracellular vesicle biomarkers	IB	EGFR+ MV count		(+): (29)		
Circulating nucleic acids	IVD	cfDNA (total)		(+): (41)		
		miR-124-3p	(+): (33)			
		miR-150	(+): (37)			
		miR-197	(+): (37)			
		miR-21	(+): (33)			
		miR-222	(+): (33)			
		miR-23a	(+): (37)			
		miR-548-5p	(+): (37)			
		TERT ctDNA mutation	(+): (42)			
	NA	miR-133a	(–): (37)			
		miR-15b	(–): (37)			
		miR-497	(–): (37)			
Circulating tumor cells	IVD	CTC	(+): (43, 44)			
Angiogenesis and inflammation related signaling molecules	NA	Ang2	(–): (59)			
		CA9	(–): (59)			
		Collagen IV	(–): (59)			
		bFGF	(–): (59)			
		MMP2	(–): (60)			
		NGAL	(–): (60)			
		PIGF	(–): (59)			
		SDF1α	(–): (59)			
		sTie2	(–): (59)			
		sVEGFR1	(–): (59)			
		sVEGFR2	(–): (59)			
		VEGF	(–): (59)			
Alterations to immune related and other circulating cell populations	IB	Mo-MDSC^i^	(+): (69)			
		NK-cells CD3+NK2GD+	(+): (68)			
		Tc-cells CD8+NK2GD+	(+): (68)			
		Treg-cells CD4+FoxP3+CD25+	(+): (68)			
	IIIC	Neutrophil to lymphocyte ratio	(+): (67)			
	NA	Lymphocytes	(–): (66)			
		NK cells	(–): (70)			
Other protein biomarkers	NA	BDNF	(–): (80)			
		B-syn-nuclein	(–): (80)			
		GFAP	(–): (80)			
		ICAM-5	(–): (80)			
		Neurogranin	(–): (80)			

*(+), positive finding; (–), negative finding; Ang, angiopoeitin; BDNF, brain derived neurotrophic factor; bFGF, basic fibroblast growth factor; CA9, carbonic anhydrase 9; CD, cluster of differentiation; cfDNA, cell-free DNA; CTC, circulating tumor cells; EGFR, epidermal growth factor receptor; FoxP3, forkhead box P3; GFAP, glial fibrillary acidic protein; ICAM-5, Intercellular adhesion molecule 5; LOE, level of evidence rating; miR, microRNA; MMP, matrix metalloproteinase; Mo-MDSC, monocyte myeloid-derived suppressor cell; MV, microvesicle; NGAL, neutrophil gelatinase-associated lipocalin; NK, natural killer; NKG2D, natural killer group 2D; PIGF, placental growth factor; SDF1α, stromal cell derived factor 1α; sTie2, soluble Tie-2; sVEGFR, soluble VEGF receptor; Tc, cytotoxic T cells; TERT, telomerase reverse transcriptase; Treg, regulatory T cells; VEGF, vascular endothelial growth factor*;

i *Mo-MDSC HLA-DR and Vannin 2 expression were combined in an index known as DR-Vannin index (DVI), see Table A9 for more details*.

## Discussion

Different biomarker subtypes present unique strengths and limitations in glioma response monitoring potential (summarized in Table 4). Exosomal markers stood out because of the host of tumor-related information accessible through a single platform, most evident in microfluidic isolation studies by Shao et al. (30, 34). In addition to promising response-related findings, micro-NMR was also able to achieve high diagnostic accuracy in differentiation of GBM and healthy controls prior to treatment (Sn = 92%, Sp = 88%, AUC = 0.95) (30). The ability to leverage tumor-specific information carried in EVs through ultrasensitive assay techniques can focus on the likely small subset of circulating EVs that are truly tumor-derived, increasing the specificity of detected changes to tumor level events. Novel assays that couple biomarker extraction and quantification steps together such as chip-based approaches may reduce turnaround times to clinical answers but, at present are limited by cost and availability. In other molecular biomarker classes, only among miRNAs did assay methodologies specifically focus on EVs as a biomarker vehicle, with other molecular biomarkers in this review isolated directly from serum or plasma samples. Wider application of EV-based assays may improve performance of alternative molecular classes.

**Table 4:** Summary of evidence for biomarker classes in this systematic review including strengths, limitations, and pathways for future clinical application.

**Biomarker class**	**Highest LOE rating for TP, SD, and/or TR**	**Highest LOE Rating for TP vs. PSP**	**Strengths**	**Limitations**	**Strategies for future clinical application**
Extracellular vesicle biomarkers	IB	IB	Specificity to glioma High sensitivity assays exist Chip based assays can couple several analytical steps reducing turnaround time	Require novel and expensive high sensitivity assays with limited availability	Application to larger cohorts with diagnostic study designs Application of exosomal protein-based assays to TP vs. PsP
Circulating nucleic acids	IB	IVD	Short half-life reflects real time tumor status qPCR techniques allow fast assay turnaround	Low case numbers for treatment monitoring studies ctDNA sensitivity limited	miRNAs: application to larger cohorts with diagnostic study designs ctDNA: higher sensitivity assays High-throughput sequencing platforms
Circulating tumor cells	IIC	IVD	Specificity to glioma	Sensitivity limited Prolonged turnaround time due to complexity in isolation	Application to larger cohorts with diagnostic study designs Improvement of isolation techniques
Angiogenesis- and inflammation-related signaling molecules	IB	NA	High methodological quality of studies ELISA techniques allow fast assay turnaround	Treatment modality specific Conflicting evidence	Diagnostic study designs Further studies during conventional CRTx
Angiogenesis-related circulating cells	IB	NA	—	Unclear definitions of key cell populations FACS-based assays increase turnaround time Treatment modality specific	Further studies to define tumor-specific cell populations
Alterations to immune-related and other circulating cell populations	IIB	IB	Evidence indicates stronger potential in TP vs. PsP May reflect pathological processes in PsP	Likely to be affected by co-existing immune modulating therapies or disorders	Further studies to define immune aberrations of interest Application to larger cohorts with diagnostic study designs
Other protein biomarkers	IB	NA	Large cohort sizes ELISA techniques allow fast assay turnaround	Limited sensitivity and specificity of initially promising candidates	Discovery studies with higher-throughput screening

*NA, not applicable*.

Early findings on CNAs in glioma are promising but face barriers. Studies on miRNA were limited by low patient numbers in response assessment analyses. Many miRNA studies were focused on diagnostic performance of biomarkers in GBM vs. healthy controls at baseline, but only small subsets of patients were followed up posttreatment, precluding assessment of diagnostic accuracy for response outcomes. With meta-analyses establishing potential of miRNAs in diagnosis of GBM vs. healthy controls enabled by well-designed studies for this question (88–91), the impetus exists for larger studies to further assess their potential role in monitoring. Circulating tumor DNA and cfDNA studies were likewise limited by small cohorts, consisting predominantly of case series. One potential advantage of ctDNA is its rapid plasma clearance, with a study in colorectal cancer demonstrating estimated plasma half-life of <1.5 h, making it theoretically more likely to reflect disease status at time of assay (92). Circulating tumor DNA levels in glioma cases are, however, among the lowest in all cancers (93). This could be mitigated through size-selected sequencing, with evidence that ctDNA occupies the 90- to 150-bp fragment size range compared with normal cfDNA at 160 bp, with size selection markedly increasing ctDNA yield in GBM and improving diagnostic accuracy vs. controls (94, 95).

The multitude of angiogenesis- and inflammation-related proteins with positive findings in this review raises significant questions. Inconsistency in findings between studies strongly suggests that these biomarkers may be markers of bioactivity of antiangiogenic agents rather than direct indicators of changes in the tumor tissue. Alterations in biomarkers at TP may reflect escape mechanisms via angiogenic cascades not inhibited by the trial agent. Alternatively, changes in such markers may only be meaningful in the context of a therapeutic agent that directly targets signaling pathways in which they are involved. An ideal angiogenic biomarker would reflect TP-associated increase in neoangiogenesis independent of treatment context. Regardless, given that antiangiogenesis-induced normalization of tumor vasculature presents a significant imaging conundrum, several markers here identified represent candidates for future study in targeted trials.

Among cellular biomarkers, in the case of CTCs, there have been progressive improvements in detection sensitivity, with the highest incidence reported at 77% of WHO II–IV patients (43). At present, however, this level of sensitivity is evidently limiting for universal application of this marker. For CECs, evidence suggests that treatment-related changes may be restricted to antiangiogenic treatment modalities. Circulating endothelial cell fluctuations may arise from direct insults from such treatment on recruitment pathways from bone marrow. A further difficulty with CECs is that there is little consensus on the definition of cell surface markers used for their isolation (15), with a strong possibility that differential phenotypic and functional properties of CECs isolated in different studies may explain heterogeneous findings in treatment monitoring. CD109 was a membrane protein selectively overexpressed in blood vessels during tumor angiogenesis in colon, lung, and breast tumors (96); thus, CD109 CECs may identify a tumor-specific population (62). Further work is required to delineate a high-fidelity CEC subpopulation that reflects GBM status.

Emerging focus on differences in immune profile as indicators of tissue level changes in GBM is promising. Pseudoprogression likely either induces or is partly driven by distinct immunological events that are reflected in the immune cell profile in peripheral blood. Taken together, Parsa et al. (68) and Soler et al. (69) suggest that assessing the balance between proinflammatory and immunosuppressive cell lineages in peripheral blood may be a clinically useful approach that deserves further exploration. The second facet of leveraging immune system changes is their potential for monitoring patients undergoing active vaccination. An ability to define responders via epitope specific assays could guide decisions on vaccine continuation or early switch to a different modality. The limitation is that epitope-specific assays are therapy specific and not generalizable.

One limitation of this review is that we did not search gray literature or include studies not available in English, which theoretically increased the risk of incomplete retrieval. There was also a paucity of studies with diagnostic design for the clinical question of interest, combined with a large proportion of studies with low methodological quality. It is emphasized that LOE gradings were based on evidence for the differential expression of biomarkers in question and not their diagnostic application. We decided against application of the GRADE criteria for diagnostic studies in appraisal, as these present significant limitations in applicability to assessing evidence on differential expression alone due to reliance on diagnostic accuracy data to ascertain inconsistency, as well as imprecision of evidence (97). The NCCN criteria allowed for broader assessment of clinical utility across a range of study designs, however, do not incorporate risk-of-bias assessment into LOE ratings to the same degree as GRADE; nor are they specifically designed to give consensus recommendations (25). It should also be noted that, as final glioma response classification is often only possible retrospectively, an appropriate interval between index test and reference standard is difficult to determine. While a 1-month cutoff was used for quality assessment in this review, it is emphasized that concurrent biomarker sampling and reference standard application are preferable to avoid misclassification biases, because of potential for disease status change in the delay between assay and imaging or biopsy.

## Conclusion

Based on this review, no biomarkers are yet ready for clinical application either as triage tests or add-on tests to the current GBM monitoring paradigm. Future studies must employ multistage verification. A marker should first be reliably detectable and specific to glioma. It follows therefore that the marker should perform strongly at distinguishing glioma-bearing patients from healthy controls. This is a precondition to diagnostic performance differentiating disease presence or progression from treatment-related phenomena, a process calling for higher levels of specificity owing to confounding pathological processes. At this stage, while circulating marker diagnosis of glioma vs. healthy controls accrues mounting experimental evidence, follow-through into monitoring phases of studies is characterized by attrition in patient numbers and methodological quality, as shown in this review. Novel sensitive assay techniques that leverage multiple bioinformatics sources in a single assay such as high-throughput microfluidic platforms or sequencing platforms likely hold the greatest potential. The possibility of using analysis of circulating immune cells to distinguish PsP from TP deserves further investigation.

## Data Availability Statement

The original contributions presented in the study are included in the article/Supplementary Material, further inquiries can be directed to the corresponding author/s.

## Author Contributions

IR wrote the protocol, screened searches and articles, performed data extraction, performed quality assessment, and wrote the manuscript. CT performed quality assessment. PG screened searches and articles, performed data extraction, verified quality assessment, and was a major contributor to the manuscript. LR formulated searches. JT was a major contributor to the protocol, methodology, and design of the paper. MH screened articles, verified data extraction, and was a major contributor the manuscript. All authors read and approved the final manuscript.

## Conflict of Interest

The authors declare that the research was conducted in the absence of any commercial or financial relationships that could be construed as a potential conflict of interest.

## Supplementary Material

Additional File 1 Search strategy, adapted QUADAS-2 scale, and adapted NCCN framework for evaluating clinical utility of biomarkers.

Additional File 2 Characteristics tables for all studies stratified by biomarker subtype.

Additional File 3 Results of quality assessment for each individual study.

## Abbreviations

+LR: positive likelihood ratio
–LR: negative likelihood ratio
AUC: area under the curve
CEC: circulating endothelial cell
CEP: circulating endothelial progenitor
cfDNA: cell free DNA
CAN: Circulating nucleic acid
CR: complete response
CRTx: chemoradiotherapy
CTC: circulating tumor cell
ctDNA: circulating tumor DNA
CTx: chemotherapy
DC: dendritic cell
DOR: diagnostic odds ratio
EV: extracellular vesicles
GFAP: glial fibrillary acidic protein
HGG: high grade glioma
lncRNA: long-non-coding-RNA
LOE: level of evidence
NA: not-applicable
miRNA: micro-RNA
Mo-MDSC: monocytic myeloid derived suppressor cells
MP: microparticles
MV: microvesicles
NK: natural killer
NMR: nuclear magnetic resonance spectroscopy
OS: overall survival
PCT: prospective controlled trial
PR: partial response
PsP: pseudoprogression
QUADAS-2: Quality Assessment of Diagnostic Accuracy Studies-2
RTx: radiotherapy
SD: stable disease
Sn: sensitivity
Sp: specificity
TE: treatment-related effect
TP: tumor progression
TR: tumor response
RANO: response assessment in neuro-oncology.
