# Supplementary material for: Blood Biomarkers of Glioma in Response Assessment Including Pseudoprogression and Other Treatment Effects: A Systematic Review
Source: Front Oncol. 2020 Aug 14;10:1191. doi: 10.3389/fonc.2020.01191 (PMC7456864; doi:10.3389/fonc.2020.01191)
Supplement: Additional File 1 — Search strategy, adapted QUADAS-2 scale, and adapted NCCN framework for evaluating clinical utility of biomarkers. [file Data_Sheet_1.docx]

**Table A1: search strategy for Ovid MEDLINE database used in this systematic review**

| 1 Glioma/ or Astrocytoma/ or Glioblastoma/ or Ganglioglioma/ or Gliosarcoma/ or Oligodendroglioma/ |
| --- |
| 2 (glioma* or ganglioglioma* or glia* tumo?r* or ganglioneuroma* or ganglioglioma* or ganglioglioneuroma* or neuroglioma ganglionare or astroglioma* or ol?goastrocytoma* or astrocytoma* or gl*oblastoma* or spongioblastoma multiforme or xanthoastrocytoma* or gliosarcoma* or ol?godendroglioma* or ol?godendrocytoma* or ol?godendroblastoma* or astroblastoma*).mp. |
| 3 ((ol?godendroglia* or astrocytic or ol?godendrocytic or glia* or neuroglia* or astroglia* or microglia* or macroglia* or tanycytic) adj (tumo?r* or neoplasm* or malignanc*)).mp. |
| 4 ((astrocytic cell or ol?godendrocytic cell or ol?godendroglia* cell or glia* cell or neuroglia* cell or astroglia* cell or microglia* cell or macroglia* cell or tanycytic cell) adj (tumo?r* or neoplasm* or malignanc*)).mp. |
| 5 (malignan* adj1 (astrocytic cell or ol?godendrocytic cell or ol?godendroglia* cell or glia* cell or neuroglia* cell or astroglia* cell or microglia* cell or macroglia* cell or tanycytic cell)).mp. |
| 6 1 or 2 or 3 or 4 or 5 |
| 7 exp Biomarkers/ |
| 8 (biomarker* or bio* marker* or clinical marker* or immun* marker* or inflammat* marker* or laboratory marker* or molecular marker* or viral marker* or surrogate end point* or surrogate endpoint* or surrogate marker* or diagnostic marker* or diagnostic indicator* or prognostic marker* or prognostic indicator* or predictive marker* or predictive indicator* or bio* indicator* or immun* indicator* or inflammat* indicator* or tumo?r marker* or cancer marker*).mp. |
| 9 exp RNA, UNTRANSLATED/ or exp RNA, LONG NONCODING/ or exp RNA, SMALL UNTRANSLATED/ or exp MICRORNAS/ or exp CIRCULATING MICRORNA/ |
| 10 (non coding RNA* or non protein coding RNA* or noncoding RNA* or untranslated RNA* or small untranslated RNA* or ncRNA* or nc RNA* or npcRNA* or npc RNA* or lincRNA* or linc RNA* or lncRNA* or lnc RNA* or sncRNA* or snc RNA* or circRNA* or circ RNA* or microRNA* or micro RNA* or miRNA* or mi RNA* or stRNA* or st RNA*).mp. |
| 11 Neoplastic Cells, Circulating/ or Endothelial Progenitor Cells/ or Circulating Tumor DNA/ or Cell-Free Nucleic Acids/ |
| 12 (circulat* cancer cell* or circulat* tumo?r cell* or circulat* neoplastic cell* or circulat* endothelial progenitor* or circulat* endothelial stem cell* or circulating EPC* or cellfree tumo?r DNA* or cell-free DNA* or circulating tumo?r DNA* or free tumo*r DNA* or circulat* DNA* or Ctdna* or cftdna* or ccfdna* or cfna* or ccfna* or cfdna* or ctdna* or ct dna* or cft dna* or ccf dna* or cf na* or ccf na* or cf dna* or ct dna* or cell-free nucleic acid* or circulat* free nucleic acid* or circulat* nucleic acid* or ctRNA* or cfRNA* or ctmiRNA* or ct mi RNA* or cirDNA or cell-free deoxyribonucleic acid* or cell-free RNA* or cf RNA* or ct RNA* or cirRNA* or cell-free ribonucleic acid* or circulat* RNA* or circulat* glio*).mp. |
| 13 Exosomes/ or Extracellular Vesicles/ or Cell-Derived Microparticles/ |
| 14 (exosome* or extracellular vesicle* or exovesicle* or microparticle* or microvesicle*).mp. |
| 15 (blood cell count* or blood cell number* or complete blood count* or white blood cell* or white blood count* or white cell count* or leu#ocyte amount* or granulocyte amount* or cd4* cell count* or cd4* count* or CD4 T cell count* or cd4-cd8 or t4-t8 or helper suppressor ratio* or suppressor helper ratio* or platelet count* or platelet number* or thrombocyt* count* or thrombocyt* number* or acidophil granulocyte count* or eosinoph* granulocyte count* or eosinoph* count* or differential cell count* or differential leu#ocyte* or differential white cell* or leu#ocyte differential* or white cell differential* or CD4* cell percentage* or CD4 lymphocyte percentage* or CD4 percentage* or CD4 T cell percentage* or CD4 T lymphocyte percentage* or CD8 cell count* or CD8 count* or CD8 T cell count*).mp. |
| 16 ((neutrophil* or monocyte* or granulocyte* or lymphocyte* or leu#ocyte* or nk cell* or natural killer cell*) adj3 (count* or number* or ratio* or percentage* or circulat*)).mp. |
| 17 7 or 8 or 9 or 10 or 11 or 12 or 13 or 14 or 15 or 16 |
| 18 exp Blood/ or exp Plasma/ or exp Serum/ or exp Blood Chemical Analysis/ or exp Hematologic Tests/ or exp Serologic Tests/ |
| 19 (circulat* or blood* or plasma* or serum* or sera or serologic* or h*ematologic*).mp. |
| 20 Liquid Biopsy/ |
| 21 (liquid biops* or fluid biops* or fluid phase biops*).mp. |
| 22 18 or 19 or 20 or 21 |
| 23 Vascular Endothelial Growth Factor A/ or Vascular Endothelial Growth Factor Receptor-1/ or Vascular Endothelial Growth Factor Receptor-2/ or Angiopoietin-2/ or Receptor, Tie-2/ or Matrix Metalloproteinase 2/ or Matrix Metalloproteinase 9/ or Matrix Metalloproteinase 10/ or Tissue Inhibitor Of Metalloproteinase-1/ or Tissue Inhibitor Of Metalloproteinase-2/ or Placenta Growth Factor/ or Platelet-Derived Growth Factor/ or Fibroblast Growth Factor 2/ or Chemokine CXCL12/ |
| 24 (cgp 39 or chi3l1 or cartilage glycoprotein 39 or chitinase-3-like protein 1 or gp 39 protein* or ylk-40 or ylk40 or YKL-40 or YKL40 or vascular endothelial cell growth factor* or VEGF or vascular endothelial growth factor* or vascular permeability factor* or vasculotropin* or Flt1 or Flt-1 or sFLT1 or sFLT-1 or proto-oncogene protein Flt or vegfr-1 or svegfr-1 or fms-like tyrosine kinase receptor* or VEGFR-2 or VEGFR2 or sVEGFR-2 or sVEGFR2 or angiopoietin 2 or ang2 or ang-2 or sTie-2 or sTie2 or tie-2 or tie2 or MMP-2 or MMP2 or collagenase type 4 or collagenase type iv or matrix metalloproteinase 2 or type iv collagenase or type 4 collagenase or MMP-9 or MMP9 or matrix metalloproteinase 9 or matrix metalloproteinase 10 or MMP-10 or MMP10 or proteoglycanase 2 or metalloproteinase-1 tissue inhibitor* or timp-1 or timp1 or tissue inhibitor of metalloproteinase-1 or tissue inhibitor of matrix metalloproteinase 1 or metalloproteinase-2 tissue inhibitor* or timp-2 or timp2 or tissue inhibitor of metalloproteinase-2 or tissue inhibitor of matrix metalloproteinase 2 or placenta* growth factor* or PLGF or placenta* derived growth factor* or PDGF or PDGFAA or platelet growth factor* or platelet derived growth factor* or thrombocyt* derived growth factor* or thrombocyt* growth factor* or basic fibroblast growth factor* or fgf-2 or fgf2 or fibroblast growth factor 2 or bFGF or chemokine c-x-c motif ligand 12 or chemokine cxcl12 or pre b-cell growth stimulating factor* or sdf-1alpha or sdf1alpha or sdf-1-alpha or sdf1-alpha or sdf1-3*a or stromal cell derived factor 1alpha or stromal cell-derived factor 1 alpha or stromal derived factor 1alpha or stromal derived factor 1 alpha or stem cell growth factor beta or scgf beta).mp. |
| 25 Interleukin-1beta/ or Interleukin 1 Receptor Antagonist Protein/ or Receptors, Interleukin-2/ or Interleukin-3/ or Interleukin-4/ or Interleukin-5/ or Interleukin-6/ or Interleukin-7/ or Interleukin-8/ or Interleukin-9/ or Interleukin-10/ or Interleukin-12/ or Interleukin-13/ or Interleukin-16/ or Interleukin-18/ |
| 26 (il-1 beta or interleukin 1beta or interleukin-1 beta or IL-1beta or IL-1B or IL1B or IL-1-B or IL1-B or interleukin 2 or il2-r or il2r or sil2-r or sil2r or colony-stimulat* factor 2 alpha or mast cell growth factor* or il-3 or interleukin 3 or mast-cell colony-stimulat* factor* or b cell growth factor* 1 or b cell growth factor I or b cell proliferat* factor* or b cell stimulat* factor 1 or b cell stimulat* factor I or bcgf-1 or bsf-1 or bsf1 or il-4 or il4 or interleukin 4 or mcgf-2 or mast cell growth factor-2 or b lymphocyte stimulat* factor 1 or eosinophil differentiat* factor* or b-cell growth factor ii or bcgf-ii or eosinophil differentiat* factor* or il-5 or il5 or interleukin 5 or t-cell replacing factor* or b cell growth factor 2 or killer helper factor* or t-lymphocyte replacing factor* or b cell differentiat* factor* or b cell stimulat* factor 2 or bsf-2 or ifn-beta 2 or il-6 or il6 or interferon beta 2 or interleukin 6 or mgi-2 or myeloid differentiat* inducing protein* or plasmacytoma growth factor* or b lymphocyte stimulat* factor 2 or beta 2 interferon* or beta2 interferon* or bsf2 or interferon beta 2 or interferon beta2 or interleukin hp1 or il-7 or il7 or interleukin 7 or lymphopoietin 1 or pre B cell growth factor* or pre b lymphocyte growth factor* or anionic neutrophil activat* peptide* or chemokine cxcl8 or granulocyte chemotactic peptide* or il-8 or il8 or interleukin 8 or neutrophil-activat* peptide* or macrophage-derived chemotactic factor* or neutrophil activati* factor* or neutrophil chemotactic factor* or CXC chemokine ligand 8 or CXCL8 or neutrophil attract* peptide* or polymorphonuclear granulocyte activat* factor* or il-9 or il9 or interleukin 9 or t-cell growth factor p40 or il-10 or il10 or interleukin-10 or cytotoxic lymphocyte maturation factor* or il-12 or il12 or interleukin 12 or natural killer cell stimulat* factor* or il-13 or il13 or interleukin 13 or il-16 or il16 or interleukin 16 or lcf factor* or lymphocyte chemoattractant factor* or ifn gamma inducing factor* or il-18 or il18 or interferon gamma inducing factor* or interleukin 18 or gamma interferon inducing factor* or interleukin 1gamma).mp. |
| 27 Macrophage Migration-Inhibitory Factors/ or Chemokine CCL3/ or Chemokine CCL4/ or Tumor Necrosis Factor-Alpha/ or Carbonic Anhydrase IX/ or Chemokine CXCL1/ or Hepatocyte Growth Factor/ or Interferon Alpha-2/ or Interferon-Gamma/ or Chemokine CXCL10/ or Chemokine CXCL9/ |
| 28 (macrophage migration inhibit* factor* or macrophage inhibit* factor* or MIF or chemokine c-c motif ligand 3 or CCL3L1 or CCL3L2 or CCL3L3 or mip-1alpha or mip1alpha or macrophage inflammat* protein 1-alpha or small inducible cytokine A3 or CC chemokine ligand 3 or CCL3 or SCYA3 or CCL4 or CCL4L1 or CCL4L2 or mip-1beta or mip1beta or mip-1 beta or macrophage inflammat* protein 1 beta or macrophage inflammat* protein 1beta2 or CC chemokine ligand 4 or SCYA4 or small inducible cytokine A4 or tumo$r necrosis factor or TNF-alfa or TNFalfa or TNF-alpha or TNFalpha or CAIX or carbonic anhydrase 9 or carbonic anhydrase ix or CA IX protein* or carbonate dehydratase 9 or carbonic anhydrase IX or protein CA IX or chemokine c-x-c motif ligand 1 or CXCL1 or growth-related oncogene alpha protein* or neutrophil-activat* protein 3 or CXCL-1 or CXC chemokine ligand 1 or SCYB1 or small inducible cytokine B1 or ifn-alpha 2 or interferon alfa-2a or interferon alfa-2b or interferon alpha-2 or interferon alpha-a or interferon-alpha2 or recombinant ifnalpha-2b or recombinant interferon alpha-2a or recombinant interferon alpha-2b or ifna2 or ifn-a2 or ifn-2-a or alpha 2 interferon or ifn alpha2 or interferon alpha ii or immune interferon or interferon-gamma or type ii interferon or gamma-interferon or ifn gamma or imunomax gamma or interferon 2 or interferon ii or IP-10 or IP10 or chemokine c-x-c motif ligand 10 or C-X-C motif chemokine 10 or 10 kda ifn gamma inducible protein* or interferon inducible protein 10 or interferon gamma inducible protein 10 or small inducible cytokine b10 or gammaip 10 protein* or CXCL10 or CXC chemokine ligand 10 or SCYB10 or chemokine c-x-c motif ligand 9 or CXCL9 or mig chemokine* or monokine induced by gamma interferon or scyb9 chemokine* or small inducible cytokine b9 or CXC chemokine ligand 9 or gamma interferon induced monokine* or SCYB9).mp. |
| 29 TNF-Related Apoptosis-Inducing Ligand/ or Chemokine CCL27/ or Chemokine CCL11/ or Chemokine CCL2/ or Chemokine CCL7/ or Chemokine CCL5/ or Granulocyte Colony-Stimulating Factor/ or Granulocyte-Macrophage Colony-Stimulating Factor/ or Stem Cell Factor/ or Nerve Growth Factor/ or E-Selectin/ |
| 30 (astroprotein* or gfa protein* or glia* fibril* acid* protein* or glia* intermediate filament protein* or g-f protein* or gf protein* or glia* filament protein* or glia* acidic fibrillary protein* or protein gf or protein gfa or GFAP or tnf related apoptosis inducing ligand* or trail protein* or TNFSF-10 or TNFSF10 or TRAIL or CTACK or CCL27 or cutaneous t-cell attracting chemokine* or small inducible cytokine a27 or CC chemokine ligand 27 or SCYA27 or CCL11 or chemokine c-c motif ligand 11 or eotaxin or MCP-1 or MCP1 or chemokine c-c motif ligand 2 or monocyte chemoattract* protein 1 or monocyte chemotactic protein 1 or monocyte chemotactic activat* factor* or CC chemokine ligand 2 or CCL2 chemokine* or CCL7 or monocyte chemoattract* protein 3 or monocyte chemotactic activat* factor 3 or monocyte chemotactic protein 3 or CC chemokine ligand 7 or chemokine c-c motif ligand 7 or MCP-3 or SCYA7 or small inducible cytokine A7 or CCL5 or rantes or CC chemokine ligand 5 or g-csf or granulocyte colony-stimulating factor* or CSF-2 or CSF-GM or Granulocyte-Macrophage Colony-Stimulating Factor* or GM-CSF or GMCSF or Histamine-Producing Cell-Stimulating Factor* or tumo$r-cell Human GM Colony-Stimulating Factor* or M-CSF or macrophage colony stimulating factor* or mast cell growth factor* or stem cell factor* or c-kit ligand* or kit ligand* or bngf or ngf-1beta or nerve growth factor* or CD62e or sCD62e or E-selectin or ELAM-1 or sELAM-1 or endothelial leukocyte adhesion molecule-1 or LECAM-2 sLECAM-2 or selectin E or leukocyte-endothelial cell adhesion molecule 2 or LECAM2 or sLECAM2 or neural?cell?adhesion?molecule* or ncam* or neural cell adhesion molecule* or brain surface protein 2 or bsp 2 protein* or cadherin N or cell adhesion molecule d2 or nerve cell adhesion molecule* or neuron glia* cell adhesion molecule* or d2 cell adhesion molecule* or N cadherin or N-CAM or NCAM or protein bsp 2 or cd56 or leu-19 or nkh-1 or NKH1 or call protein* or caml1 or cell adhesion molecule l1 or cell surface glycoprotein l1 or f11 glycoprotein* or l1 cell adhesion molecule* or l1cam or ngf inducible glycoprotein* or nile glycoprotein* or nile protein* or nerve growth factor inducible large external glycoprotein* or neural adhesion molecule l1 or neural cell adhesion molecule l1 or CAM L1 or glycoprotein NILE or L1 CAM).mp. |
| 31 exp Genomics/ or Transcriptome/ or Gene Expression Profiling/ or Metabolomics/ |
| 32 (epigenetic* or epigenomic* or genomic* or proteomic* or proteogenomic* or metabolomic* or metabolic profil* or gene expression* or transcriptom* or transcription* expression analys* or mRNA or transcription* profil* or expressed gene*).mp. |
| 33 23 or 24 or 25 or 26 or 27 or 28 or 29 or 30 or 31 or 32 |
| 34 exp animals/ not humans.sh. |
| 35 (newborn* or neonat* or infant* or infancy or preschool* or pre-school* or child* or adolescen* or teen* or p*ediatric*).mp. |
| 36 (disease?status* or response* or regression* or reduction* or stable?disease* or progress* or relapse* or recur* or grow* or pseudo$progression or radio$necrosis or radiation?necrosis or treatment?effect* or treatment?related?brain?injur* or radiation?induced?brain?injur* or radiation?effect* or radiation?injur* or pseudo$lesion* or pseudo$response* or RANO or MacDonald* or WHO criteria or RECIST).mp. |
| 37 Treatment outcome/ or neoplasm recurrence, local/ or disease progression/ or radiation injuries/ or response evaluation criteria in solid tumors/ |
| 38 36 or 37 |
| 39 6 and 22 and 38 and (17 or 33) |
| 40 39 not (34 or 35) |
| 41 limit 40 to ("all infant (birth to 23 months)" or "all child (0 to 18 years)") |
| 42 40 not 41 |
| 43 limit 42 to english language |
| 44 limit 43 to (comment or editorial or letter or news) |
| 45 43 not 44 |
| 46 limit 45 to ed=19900101-20190818 |

**Table A2: Adapted QUADAS-2 scale for quality assessment of included studies across 4 domains**

| Domain | 1. Patient selection | 2. Index test | 3. Reference Standard | 4. Flow and timing |
| --- | --- | --- | --- | --- |
| Signalling questions  Yes/No/Unclear | Was a consecutive or random sample of patients enrolled?  Were the inclusion and exclusion criteria specified appropriate? | Was the assay method valid and reliable?  Were the index test results interpreted without knowledge of the results of the reference standard? | Was the reference standard likely to correctly classify the target condition?  Were the reference standard results interpreted without knowledge of the results of the index test? | Was there an appropriate interval between index test and reference standard?  Did all patients receive the same reference standard? |

**Table A3: Adapted framework based on the National Comprehensive Cancer Network framework for evaluating the clinical utility of tumour markers in oncology**

| Clinical Trial Design | Patients and patient data | Specimen collection, processing and archival | Validational studies | Level of evidence |
| --- | --- | --- | --- | --- |
| PCT on tumor marker objective | Prospectively enrolled, treated, and followed in PRCT | Specimens collected, processed, and assayed for specific marker in real time | None required | IA |
| PCT with tumor marker secondary objective | Prospectively enrolled, treated, and followed in clinical trial | Specimens collected, processed, and assayed using operating procedures not specifically defined for biomarker question | One or more with consistent results | IB |
|  |  |  | None or inconsistent results | IIB |
| Prospective observational registry | Prospective enrolment in registry/biobank |  | 2 or more with consistent results | IIC |
|  |  |  | None or 1 with consistent results or inconsistent results | IIIC |
| No prospective aspect to study | Retrospective chart review | Specimens collected, processed, and archived with no prospective SOPs | N/A | IVD |
