# Supplementary material for: Blood Biomarkers of Glioma in Response Assessment Including Pseudoprogression and Other Treatment Effects: A Systematic Review
Source: Front Oncol. 2020 Aug 14;10:1191. doi: 10.3389/fonc.2020.01191 (PMC7456864; doi:10.3389/fonc.2020.01191)
Supplement: Additional File 2 — Characteristics tables for all studies stratified by biomarker subtype. [file Data_Sheet_2.docx]

**Table A4. Extracellular vesicles (EVs)**

| **Study** | **Biomarker (Source)** | **Assay technique** | **Sample size Histology** | **Treatment** | **Reference Standard** | **Sampling points** | **Results** |
| --- | --- | --- | --- | --- | --- | --- | --- |
| **Standard treatment** |  |  |  |  |  |  |  |
| Osti et al, 2018 | EVs (Plasma) | NTA | 23 GBM | Surgery + TMZ ± RTx ± repeat surgery | MRI at TP (RANO criteria) | Post-op; at TP | **TP vs post-op**: higher concentration at TP (p=0.028) |
| Sartori et al, 2013 | Circulating MPs:  GFAP+/TF-, GFAP-/TF+ and GFAP+/TF+ subsets (Blood) | FACS | 23 GBM | Surgery + RTx + TMZ | MRI at CRTx end, 3m post-CRTx (Macdonald’s criteria) | CRTx end & 3m post-CRTx | Comparison of exosome concentration 3m post-CRTx vs CRTx end  **TP at 3m post CRTx**: higher GFAP+/TF+ (p=0.026)  no change in GFAP-/TF+, GFAP+/TF- (p>0.05)  **SD at 3m post CRTx**: no change in GFAP+/TF+, GFAP-/TF+ or GFAP+/TF- |
| **Immunotherapy** |  |  |  |  |  |  |  |
| Galbo et al, 2018 | CD9+ exosomes:  GFAP+, SVN+ and SVN+/GFAP+ subsets (Serum) | FACS | 8 rWHO III-IV | 4x SVN vaccinations | MRI at TP (Criteria NOS) | Pre-, 8w post-vaccine | Comparison of percentage change in exosome incidence at 8w post- vs pre-vaccine  **Early (<25.1w) vs Late TP (>25.1w):** Increased SVN+ (p=0.0299), increased GFAP+/ SVN+ (p=0.0225); no difference for GFAP+ |
| **Combination therapy/others** |  |  |  |  |  |  |  |
| Koch et al, 2014 | Annexin V+/EGFR+ MV (Plasma) | FACS | 11 GBM | RTx ± TMZ ± Bev ± repeat surgery | MRI – pre-CRTx, 1, 3, 6, 12 and 24m post CRTx (RANO criteria) | pre-CRTx; 1, 3, 6, 12 & 24m post- CRTx; | **TP vs PsP**: Higher counts in samples at TP (p=0.014) |
| Shao et al, 2012 | MVs, EGFR, EGFRvIII, PDPN, IDH1-R132H proteins (Plasma) | microfluidic chip + µNMR | 12 GBM | RTx + TMZ ± trial therapy (Cediranib, Cabozantinib, Bev or Vandetanib) | MRI & clinical data (Criteria NOS) | Pre-CRTx, CRTx end | **TR vs non-TR**: Higher drug efficacy index in TR at CRTx end^i^ (p<0.005) |

**Table A4 legend:** µNMR: micro-nuclear magnetic resonance; Bev: bevacizumab; CD9: cluster of differentiation-9; CRTx: chemoradiation; EGFR: epidermal growth factor receptor; EGFRvIII: EGFR variant III mutation; EVs: Extracellular vesicles; FACS: fluorescence-activated cell sorting; GBM: glioblastoma; GFAP: Glial fibrillary acidic protein; IDH-R132H: cytosolic isocitrate dehydrogenase 1 mutation; m: months; MV: microvesicles; MP: microparticles, NTA: nanoparticle tracking analysis; PDPN: podoplanin, post-op: post-operative, PsP: pseudoprogression; RANO: response assessment in neuro-oncology; RTx: radiotherapy; rWHO: recurrent WHO grade as specified; SD: stable disease; SVN: survivin; TE: treatment effect; TF: tissue factor, TMZ: temozolomide; TP: tumour progression; TR: tumour response; WHO-I, II, II or IV: World Health Organisation grade I, II, III, or IV glioma

i: drug efficacy index was calculated as inverse of tumour progression index (TPI) where TPI = 1-response index, where response index = [(1−Δn) + Σ(1−Δξk)] / N, where 1−Δn and 1−Δξk are the relative changes in glioma MV numbers and MV biomarker expression levels respectively, and N is the total number of markers monitored (k, biomarkers used).

**Table A5. Exosomal and free Circulating Nucleic Acids (CNAs)**

| **Study** | **Biomarker (Source)** | **Assay technique** | **Sample size Histology** | **Treatment** | **Reference Standard** | **Sampling points** | **Results** |
| --- | --- | --- | --- | --- | --- | --- | --- |
| **Standard treatment** |  |  |  |  |  |  |  |
| Cordova et al, 2019* | C228T/C250T mutated TERT promoter ctDNA (Plasma) | ddPCR | 7 IDH*wt* GBM | Surgery + RTx | MRI post-Rx/at suspected TP (Criteria NOS) | Pre-op, post-op; 0, 1, 3 & 6m post-RTx | **TP vs pre-op:** ctDNA+ at TP vs ctDNA- preop (n=2)  **TP vs PsP:** 3/4 pts ctDNA levels on serial samples correlated with eventual clinical outcome  **SD vs pre-op:** declining ctDNA levels on serial samples (n=1) |
| Ilhan-Mutlu et al, 2012 | miR-21 (Plasma) | qRT-PCR | 1 GBM | Surgery + CRTx | MRI (Criteria NOS) & histopathology | During CRTx 8w pre-MRI | **TP**: Concentration increase 8w before TP |
| Lan et al, 2018 | Exosomal miR-301a (Serum) | qRT-PCR | 7 GBM | Surgery + CRTx NOS | Not specified | 2w post-op, TP | **TP vs post-op:** Expression levels increased at TP (p<0.01) |
| Santangelo et al, 2018 | Cumulative exosomal miRNA panel: miR-21, miR-124-3p and miR-222 (Serum) | qRT-PCR | 2 GBM | Pt1: Surgery + CRTx NOS, Pt2: Surgery + TMZ/RTx | MRI, DSC MRI & MRS at 3 & 5m post-op  (Criteria NOS) | Early post-op, 3m post-op | Cases with equivocal new enhancing MRI lesion at 3m post-op  **PsP:** Cumulative expression level decreased at 3m, prior to MRI confirmation at 5m (n=1)  **TP:** Cumulative expression level increased at 3m (n=1), prior to MRI confirmation at 5m (n=1) |
| Shao et al, 2015 | Exosomal APNG, MGMT mRNA (Serum) | iMER | 7 GBM | TMZ | MRI serially during & post-CTx (RANO criteria) | Pre-CTx, serially during & post-CTx | Comparison of copy number at response assessment vs baseline  **TR at time of sample:** Copy number reduced (2 samples)  **SD at time of sample:** Copy number stable (4 samples)  **TP at time of sample:** Copy number increased (5 samples) |
| Siegal et al, 2016 | miR-10b, miR-21 (Serum) | qRT-PCR | 13 HGG | TMZ + RTx | MRI serially during & post-CRTx (RANO criteria) | Pre-CRTx, serial post-CRTx (within 14d of MRIs) | **TP vs SD vs PR vs CR**: No correlation between fold change in expression of either miRNA (post-CRTx vs pre-CRTx) and response across serial samples |
| Swellam et al, 2019 | miRNA-221 miRNA-222 (Serum) | qRT-PCR | 20 GBM | Surgery + RTX + TMZ | MRI + clinical data post-CRTx (Criteria NOS) | Post-CRTx | **CR vs PR vs SD vs TP:** Sequentially higher expression levels between response groups (miR-221: p<0.0001, miR-222: p=0.02) |
| Yang et al, 2013 | MiRNA panel miR-15b, -23a, -133a, -150, -197, -497, -548-5p (Serum) | qRT-PCR | 55 WHO II, 45 WHO III, 33 WHO IV AC;  11 astrogliosis | Surgery, RTx | Histopathology | Pre-op | **Active disease vs PsP:** Concentrations of miR-23a, miR-150, miR-197 and miR-548b-5p lower for WHO II, III, IV vs PsP (p<0.05); no difference for miR-15b, miR-133a, miR-497  **Diagnostic performance for PsP vs malignant astrocytoma (WHO II-IV) (AUC with 95%CI)**  miR-23a: AUC = 0.798 (0.692-0.904)  miR-150: AUC = 0.988 (0.971-1.000)  miR-197: AUC = 0.930 (0.877-0.983)  miR-548b-5p: AUC = 0.800 (0.650-0.951)  Four miR Panel: 0.950 (0.902-0.998) |
| Yue et al, 2016 | miR-205 (Serum) | qRT-PCR | 6 GBM | Surgery + CRTx NOS | Not specified | 2w post-op, TP | **TP vs 2w post-op:** Expression levels decreased at TP (p<0.01) |
| **Anti-angiogenic therapy** |  |  |  |  |  |  |  |
| Siegal et al, 2016 | miR-10b, miR-21 (Serum) | qRT-PCR | 15 HGG | Bev | MRI serially during & post-Rx (RANO criteria) | Pre-Rx, serial post-Rx (within 14d of MRIs) | **TP vs SD vs PR vs CR**: No correlation between fold change in expression of either miRNA (post-Rx vs pre-Rx) and response across serial samples |
| **Combination therapy/others** |  |  |  |  |  |  |  |
| Faiq et al, 2015* | ctDNA (whole blood) | Guardant 360 ctDNA panel | 40 GBM | Not specified | Not specified | <30 or >30d pre-TP | ctDNA mutation frequency  **< 30 days prior to TP:** Frequency 9/16 (56%)  **> 30 days prior to TP:** Frequency 5/24 (21%)  **Diagnostic performance of presence of genetic alteration for TP in <30d vs >30d**  Sn = 56% Sp = 79% +LR = 2.70 –LR = 0.55 DOR = 4.91 |
| Faria et al,2018 | cfDNA (serum) | Fluorimetry | 3 GBM | Intranasal perillyl alcohol | MRI post Rx, at TR/TP (Criteria NOS) | Pre-Rx, post-Rx, at TR/TP | **TR:** cfDNA levels decreased at TR vs pre-treatment (n=2)  **TP:** cfDNA levels increased at TP vs at treatment end (n=1) |
| Noroxe et al, 2019 | cfDNA (plasma) | Fluorimetry | 8 GBM | RTX + TMZ ± PDL-1i/placebo | MRI at TP (Criteria NOS)  Equivocal for TP:  ± FET/PET ± MDM | Every 1-2w during CRTx, serially post-CRTx | **TP:** mean cfDNA highest at TP vs other timepoints (n=4)  In 3/4 cases cfDNA increased prior to or at TP vs preceding timepoint  **SD/TR:** In 3/4 cases cfDNA remained stable or decreased vs preceding timepoint  In cases of equivocal new enhancing lesion:  **TP:** cfDNA rise prior to MRI confirmation (n=1)  **PsP:** cfDNA decrease (n=1) or cfDNA stable (n=1) prior to MDM conclusion |

**Table A5 legend:** +LR = positive likelihood ratio; -LR = negative likelihood ratio; AC: astrocytoma; APNG: alkylpurine DNA n-glycosylase; AUC: area under the curve; Bev: bevacizumab; CE: Contrast Enhanced; cfDNA: cell-free DNA; CI: confidence interval; CNA: Circulating nucleic acids; ctDNA: circulating tumour DNA; CR: complete response; CRTx: chemoradiation; CTx: chemotherapy; d: days; ddPCR: droplet digital polymerase chain reaction; DOR: diagnostic odds ratio; DSC: dynamic susceptibility contrast; EGFR: epidermal growth factor receptor; FET/PET: Fluoro-O-(2) fluoroethyl-l-tyrosine/positron emission tomography; GBM: glioblastoma; IDH*wt*: Isocitrate dehydrogenase wildtype; iMER: immuno-magnetic exosome RNA; m: months; MDM: multidisciplinary meeting; MGMT: O-6-methyl-guanine-DNA-methyl-transferase; miRNA/miR: microRNA; MRI: Magnetic resonance imaging; MRS: magnetic resonance spectroscopy; NOS: not otherwise specified; PD: progressive disease; PD-L1i: Programmed death-ligand 1 inhibitor; PR: partial response; pre-op: preoperative; post-op: postoperative; PsP: pseudoprogression; qRT-PCR: quantitative reverse transcription polymerase chain reaction; RANO: Response Assessment in Neuro Oncology; RTx: radiotherapy; Rx: treatment; SD: stable disease; Sn = sensitivity; Sp= specificity; TERT: telomerase reverse transcriptase; TMZ: temozolomide; TP: tumour progression; TR: tumour recurrence; w: weeks; WHO-I, II, II or IV: World Health Organisation grade I, II, III, or IV glioma

* = abstract

**Table A6. Circulating Tumour Cells (CTCs)**

| **Study** | **Biomarker** | **Assay technique** | **Sample size Histology** | **Treatment** | **Reference Standard** | **Sampling points** | **Results** |
| --- | --- | --- | --- | --- | --- | --- | --- |
| **Standard treatment** |  |  |  |  |  |  |  |
| Gao et al, 2016 | CTCs (blood) | Polyploid chromosome 8 FISH | 5 HGG | Surgery + RTx + TMZ +/- repeat surgery | MRI, rCBV ± histopathology post CRTx  (Criteria NOS) | Post-CRTx | At time of new enhancing lesion post-CRTx:  **TP**: CTC+ with increased rCBV and subsequent histological confirmation (n=2); CTC+ with reduced rCBV and subsequent histological confirmation (n=1)  **PsP**: CTC- with reduced rCBV and serial MRI confirmation (n=1) |
| Macarthur et al, 2014 | CTCs (blood) | hTERT probe | 2 HGG | Surgery + RTx +/- CTx NOS | MRI, rCBV post-RTx  (Criteria NOS) | Pre- & post-RTx | CTC concentration at time of new enhancing lesion post-RTx  **TP**: CTC concentration increased vs pre-RTx (n=1) prior to rCBV confirmation  **PsP:** CTC reduction vs pre-RTx (n=1) prior to MRI confirmation |
| Stragliotto et al, 2016* | CTCs (blood) | Serial culture | 4 GBM | Surgery ± CTx NOS | MRI + clinical data (Criteria NOS) | Pre-CTx, serial during CTx | **TP:** high CTC yield in serial sampling during CTx (n=3)  **SD:** low CTC yield in serial sampling during CTx (n=1) |
| Sullivan et al, 2014 | CTCs (blood) | Microfluidic device: A2B5, c-MET, EGFR, SOX2, Tubulin beta-3 antibodies | 33 GBM | Surgery + Rx NOS | Not specified | Serial post-op | **TP vs SD:** Median CTC counts sampled at multiple post-op timepoints higher in TP (p<0.001) |

**Table A6 legend:** A2B5: cell surface ganglioside epitope; c-MET: tyrosine protein kinase Met; CRTx: chemoradiation; CTC: circulating tumour cells; CTx: chemotherapy; EGFR: epidermal growth factor receptor; FISH: Fluorescent in situ hybridisation; GBM: glioblastoma, HGG: High-grade glioma, hTERT: human telomerase reverse transcriptase; NOS: note otherwise specified; PD: progressive disease; pre-op: preoperative; post-op: postoperative; PsP: pseudoprogression; rCBV: relative cerebral blood volume; RN: radiation necrosis; RTx: radiotherapy; SD: stable Disease; SOX2: Sex Determining Region Y-Box Transcription Factor 2; TMZ: temozolomide; TP: tumour progression

*: abstract

### **Table A7. Angiogenic and inflammatory signalling molecules**

| **Study** | **Biomarker** | **Assay technique** | **Sample size Histology** | **Treatment** | **Reference Standard** | **Sampling points** | **Results** |
| --- | --- | --- | --- | --- | --- | --- | --- |
| **Standard treatment** |  |  |  |  |  |  |  |
| Iwamoto et al, 2011 | MMP-9 (serum) | ELISA | 41 LGG, 105 AG, 197 GBM | Surgery + RTx + TMZ (n=58 patients) others NOS | MRI at enrolment, every 2-3m  (Macdonald criteria) | At enrolment; every 2-3m (within 30 days of matched MRI) | **CR vs PR/SD/TP:** No difference in MMP-9 levels using MRI-matched samples in LGG, GBM and AG |
| Kesari et al, 2008 | bFGF, endostatin, TSP-1, VEGF (serum) | ELISA | 30 WHO IV | TMZ + thalidomide + celecoxib | MRI at 2m (modified Macdonald criteria) | Pre-CTx, 2m post-CTx start | **TP vs PR/SD:** No difference in absolute change and % change in level at 2m vs baseline for all biomarkers |
| Lin et al 2009 | IGFBP-2 (plasma) | ELISA | 15 GBM | Surgery + RTx ± chemotherapy NOS | MRI at 2 cycles, at TP (Criteria NOS) | Post-2 cycles CTx, at TP | **TP vs at 2-cycles CTx:** IGFBP-2 concentration increased at TP (p<0.001) |
| Lin et al, 2013 | TIMP1 (plasma) | ELISA | 88 WHO II-IV | Surgery, CRTx NOS | MRI at TP (Criteria NOS) | At 2 cycles CRTx, at TP | **TP vs at 2-cycles CTx:** No difference in TIMP-1 concentration (p>0.05) |
| **Immunotherapy** |  | | | | | | |
| Pellegatta 2013 | IL-12 (serum) | ELISA | 2 rGBM | DC Vaccine | MRI at 6m post-vaccine start  (McDonald criteria) | Pre-vaccine start, at 22w post-vaccine start (7^th^ vaccine) | **SD at 6m:** IL-12 level not changed at 22w (n=1)  **TP at 6m:** IL-12 level not changed at 22w (n=1) |
| **Anti-angiogenic therapy** |  |  |  |  |  |  |  |
| Batchelor et al, 2010 | Ang1, Ang2, bFGF, IL-1β, IL-6, IL-8, MMP-2, MMP-10, PlGF, SDF-1α, sTie-2, sVEGFR-1, sVEGFR-2, TGF-α, VEGF (plasma) | ELISA | 31 rGBM | Cediranib | MRI, PWI/DSC MRI, DCE MRI every 28d post Rx start (3D volumetric program + 2D Macdonald criteria) | Pre-Rx, every 28d post Rx start | Samples matched with MRI:  **TP:** associated with increased sVEGFR1, sTie2, SDF1α levels (p<0.05)  **PR:** associated with higher PIGF and lower sTie2 & bFGF levels (p<0.05)  No associations for other biomarkers |
| de Groot et al 2011 | bFGF, CA9, IL-6, IL-8, MIP-1β, MMP-9, PIGF, SCGFβ, SDF1α, sVEGFR2, TIMP1, VEGF (plasma) | ELISA,  LINCOplex, multiplex | 26 rGBM | Aflibercept | MRI at 28d (Macdonald criteria) | Pre-Rx & 28d post-Rx start | **TP vs SD vs PR at 28d:** Increase in MMP9 (p=0.07) and TIMP1 (p=0.03) at 28d associated with worse response  No significant change for the other biomarkers |
| Labussière et al, 2016 | Ang2, PIGF, sTie2, VEGF (plasma) | ELISA | 70 rGBM | Bev | MRI at TP (RANO criteria) | Post-2 cycles Rx, post-4 cycles Rx, at TP | **TP vs 2 cycles:** Ang2 concentration increased at TP (p=0.0137)  **TP vs 4 cycles:** Ang2 concentration increased at TP (p=0.0123)  No difference for other biomarkers |
| Pace et al, 2018 | D-dimer, F1+2, FVIII, PAI-1, TAT, vWF, (plasma); VEGF (serum) | Immunoassay | 49 rHGG | Bev | MRI at 3m (RANO criteria) | pre-2^nd^ cycle Rx, pre-3^rd^ cycle Rx | **PR vs SD vs TP at 3m:** vWF levels higher at pre-3^rd^ cycle timepoint with worsening response (p=0.02)  **PR vs SD/TP at 3m:** vWF levels lower at pre-2^nd^ and pre-3^rd^ cycle timepoints in PR (p=0.02)  FVIII levels lower at pre3rd cycle in PR (p=0.045)  No differences between response outcomes for other biomarkers |
| **Combination therapy/others** |  |  |  |  |  |  |  |
| Chinnaiyan et al, 2012 | Angiogenin , Angiostatin, IGF-1, IGF-1SR, IGF-2, IGFBP-1, IGFBP-2, IGFBP-3, IGFBP-4, IGFBP-5, IGFBP-6, PDGF-AA, PDGF-BB, PDGFR-α, PDGFR-β, sVEGFR2, sVEGFR3, VEGF, VEGF-D, VEGF-C (plasma) | Human antibody array | 10 recurrent WHO IV | Bev + irinotecan+ vorinostat | MRI at TP (Criteria NOS) | Pre-Rx, every 2nd cycle until TP | **TP vs pre-Rx:** Decreased PDGF-AA levels at TP (p=0.015); no change for all other biomarkers |
| Eoli et al, 2012* | bFGF, G-CSF, IL-8, IL-12, IL-13, IL-17, MIP-1β, PDGF-β, VEGF (plasma) | Bio-Plex® | 63 rHGG | Bev + irinotecan | MRI at 8w (modified RANO criteria) | Pre-Rx, every 8w during Rx | **TP vs CR/PR at ≤ 8w:** VEGF concentration decreased at 8w vs pre-Rx in CR/PR (p=0.001), remained stable at 8w vs pre-Rx for TP  All other biomarkers remained stable regardless of response |
| Gomes et al, 2011* | IL-8, PDGF, VEGF (plasma) | ELISA | 83 rGBM | Monoterpene perillyl alcohol | Clinical + histopathology ± MRI (RANO criteria) | Baseline, every 3m during Rx | **TR:** VEGF, IL-8, PDGF levels decreased from baseline in patients with TR (p<0.01)  **TP:** changes in biomarkers often preceded TP (data not shown) |
| Lee et al 2015 | Ang2, bFGF, CA9, Collagen IV, PIGF, SDF-1α, sTie2, sVEGFR1, sVEGFR2, VEGF (plasma) | ELISA | 45 GBM | TMZ + RTx + vandetanib | MRI every 8w (modified McDonald’s criteria) | Pre-Rx, every 2^nd^ 28d cycle maintenance phase | Samples matched with MRI:  **CR vs PR vs SD vs PsP vs TP:** no association between change in any biomarker vs pre-Rx level and response at time of MRI* |
| Shehan et al, 2016* | MMP-2, NGAL (serum) | ELISA | 13 GBM | Surgery + RTx + TMZ ± Bev | MRI every 8w or at suspected TP (RANO criteria) | Early post-op, every 8w | Samples matched to MRI  **PsP vs TP:** No difference in longitudinal change in MMP2 or NGAL expression at MRI vs early post-op |
| Tabouret et al, 2015 | MMP-2, MMP-9 (plasma) | ELISA | 41 rHGG | Bev + irinotecan | MRI at TP (McDonald criteria)* | Pre-Rx, then every 14 days until TP | **TP vs last point prior to TP:**  MMP2 levels increased (p=0.033); no change for MMP9 |

**Table A7 legend:** AG: anaplastic glioma; Ang: angiopoietin; Bev: bevacizumab; bFGF: basic fibroblast growth factor; CA9: carbonic anhydrase 9; CR: complete response; CRTx: chemoradiotherapy; CTx: chemotherapy; d: days; DC: dendritic cell; DCE: dynamic contrast enhanced; DSC: dynamic susceptibility contrast; ELISA: enzyme linked immunosorbent assay; F1+2: prothrombin fragment 1+2; FVIII: factor VIII; GBM: glioblastoma; G-CSF: granulocyte-colony stimulating factor; IGF: insulin like growth factor: IGFBP: Insulin Like Growth Factor Binding Protein; IL- interleukin; LGG: low grade glioma; NGAL: neutrophil gelatinase-associated lipocalin; NOS: not otherwise specified; m: months; MIP-1β: macrophage inflammatory protein-1β; MMP: matrix metalloproteinase; PAI-1: plasminogen activator inhibitor-1; PDGF: platelet derived growth factor; PDGFR: platelet derived growth factor receptor; PIGF: placental growth factor; PR: partial response; PsP: pseudoprogression; PWI: perfusion weighted imaging; RTx: radiotherapy; r: recurrent; Rx: treatment; SCGFβ: serum stem cell growth factor β; SD: stable disease; SDF-1α: stromal cell derived factor 1α; sTie-2: soluble Tie-2; sVEGFR: soluble VEGF receptor; TAT: thrombin-antithrombin complexes; TGF- α: transforming growth factor α; TIMP1: tissue inhibitor of matrix metalloproteinase 1; TMZ: temozolomide; TP: tumour progression; TR: tumour response; TSP-1: thrombospondin 1; VEGF: vascular endothelial growth factor; vWF: von-Willebrand factor; w: weeks; WHO I, II, II or IV: World Health Organisation grade I, II, III, or IV glioma

*= abstract

### **Table A8. Angiogenesis related circulating cells**

| **Study** | **Biomarker** | **Assay technique** | **Sample size Histology** | **Treatment** | **Reference Standard** | **Sampling points** | **Results** |
| --- | --- | --- | --- | --- | --- | --- | --- |
| **Standard treatment** |  |  |  |  |  |  |  |
| Cuppini et al 2013 | CD109+, CECs, CECs, HPCs, PPCs, viable CECs (blood) | FACS | 14 rGBM | TMZ / Fotemustine | MRI at 8w (RANO criteria) | Pre-Rx, 8w post-Rx | **PR/SD at 8w:** no change in all cell counts at 8w |
| Greenfield et al 2009 | CPCs (blood) | FACS | 1 HGG | Surgery + adjuvant therapy NOS | Not specified | Post-op, at TP | **TP vs post-op:** CPC count increased at TP |
| **Anti-angiogenic therapy** |  |  |  |  |  |  |  |
| Batchelor et al 2007 | Viable CEC (blood) | FACS | 16 rGBM | Cediranib | MRI at 28d, 56d then every 60d (volumetric analysis) | Pre-Rx; 28d post-Rx start, then every 28d | **TP:** associated with concurrent increase in CEC count on MRI matched samples (p=0.0347) |
| Batchelor et al, 2010 | CPCs (blood) | FACS | 31 rGBM | Cediranib | MRI, PWI/DSC MRI, DCE MRI every 28d post Rx start (3D volumetric program + 2D Macdonald criteria) | Pre-Rx, every 28d post Rx start | Samples matched with MRI:  **TP:** no association with change in CPC count  **PR:** no association with change in CPC count |
| Cuppini et al 2013 | CECs, viable CEC, CD109+ CECs, HPC, PPC (blood) | FACS | 17 rGBM | Bev | MRI at 8w (RANO criteria) | Pre-Rx, 8w post-Rx | **PR/SD at 8w:** CD109+ CEC counts decreased at 8w (p=0.001), no change for other cell types |
| Galanis et al 2013 | CECs (blood) | FACS | 49 rGBM | Bev + sorafenib | MRI at trial end (RANO criteria) | Baseline, at TP/withdrawal/ removal | **TP vs TR/SD at trial end:** CEC counts increased in TP cases (p=0.022) |
| **Combination therapy/others** |  |  |  |  |  |  |  |
| Cuppini et al 2013 | CECs, viable CEC, CD109+ CECs, HPC, PPC (blood) | FACS | 56 rGBM | Bev + irinotecan | MRI at 8w (RANO criteria) | Pre-Rx, 8w post-Rx | **TP at 8w:** no change in all cell counts at 8w  **PR/SD at 8w:** CEC, viable CEC, CD109+ CEC, HPC, PPC counts decreased at 8w (p=0.05, p=0.02, p=0.0002, p=0.008) |

**Table A8 legend:** CEC: circulating endothelial cell; CD: cluster of differentiation; CPC: circulating progenitor cell; d: days; FACS: fluorescence-activated cell sorting; GBM: glioblastoma; HGG: high grade glioma; HPC: haematopoetic progenitor cells; post-op: postoperative; PPC: progenitor perivascular cell; PR: partial response; pre-op: preoperative; r: recurrent; RANO: response assessment in neuro-oncology; SD: stable disease; TMZ: temozolomide; TP: tumour progression; w: weeks

**Table A9. Changes to immune-system and other circulating cell lines**

| **Study** | **Biomarker** | **Assay technique** | **Sample size Histology** | **Treatment** | **Reference Standard** | **Sampling points** | **Results** |
| --- | --- | --- | --- | --- | --- | --- | --- |
| **Standard treatment** |  |  |  |  |  |  |  |
| Hassan et al, 2017* | TLC (blood) | FACS | 45 GBM | Surgery + RTx/TMZ | MRI at suspected TP + histopathology (RANO criteria) | After CRTx | **PsP vs TP:** No difference in TLC counts |
| Huang et al 2019 | NLR (blood) | Not specified | 75 HGG | Surgery + RTx/TMZ | Multimodal MRI/MDT consensus at 4w post-CRTx, 2 & 5 cycles adjuvant CTx (Criteria NOS) | Pre-CRTx, at TP or CRTx completion | **PsP vs TP**: NLR decrease^i^ associated with PsP (p=0.022).  NLR increase^i^ not associated with PsP.  **Diagnostic performance for NLR decrease in PsP vs TP:**  Sn:59%, Sp: 67%, +LR:1.85, -LR: 0.60 DOR = 3.08 |
| Parsa et al 2010* | NK-cells CD3+NK2GD+ (activated), Tc CD8+NK2GD+ (activated), Treg CD4+FoxP3+  CD25+, | FACS | 37 recurrent glioma, 8 PsP | CRTx + surgery | Histopathology | Pre-op | **TP vs PsP:** Treg higher, activated Tc and activated NK cells lower in TP cases |
| Soler et al, 2017 | Mo-MDSC – DVI^ii^ (blood) | Immuno-  staining | 13 new GBM, 5rGBM, 6 PsP | Surgery + TMZ/RTx ± repeat surgery | Histopathology | Pre-op | **Active disease vs PsP:** higher % of Mo-MDSC cells HLA-DR^neg/low^ (p=0.0004)  lower % of Mo-MDSC cells VNN2+ (p=0.0002) higher ratio of HLA-DR^neg/low^/VNN2+ Mo-MDSCs (DVI) (p=0.0002)  **TP vs PsP:** DVI higher in TP (rGBM) vs PsP (p=0.0004) |
| Sturla et al, 2013* | NK cells (blood) | Not specified | 10 HGG | Paclitaxel Poliglumex + RTx/TMZ | MRI every 2m (modified McDonald criteria) | Weekly during CRTx, monthly during adjuvant TMZ | **PsP then resolution:** Trend increase in NK cell counts 1 month prior to PsP and then decreased with resolution (n=4, p=ns)  **PsP then TP:** Trend increase in NK cell counts 1 month prior to PsP, further increase prior to TP (n=6, p=ns) |
| Anti-angiogenic |  | | | | | | |
| Pace et al, 2018 | Lymphocytes, MPV, Neutrophils, PDW, Platelets (whole blood) | FACS | 49 rHGG | Bev | MRI at 3m (RANO criteria) | pre-2^nd^ cycle Rx, pre-3^rd^ cycle Rx | **PR vs SD vs TP:** no differences between response outcomes at all timepoints (p>0.05) |
| **Immuno**  **therapy** |  |  |  |  |  |  |  |
| Okada et al 2011 | Mononuclear cells IFN-γ (blood)  CD8+ T cells – antigen specific (blood) | IFN-γ ELISpot assay, Tetramer assay | 19 rWHOIII-IV | DC vaccine | MRI at 9w (McDonald criteria) | At 9w post-vaccine start | Samples matched with MRI  **TP:** 5/6 ELISpot+ and 1/4 tetramer+ at 9w^iii^ **PR:** 0/2 ELISpot+ and 1/2 tetramer at 9w+  **SD:** 5/11 ELISpot+ and 6/11 tetramer at 9w+ |
| Pellegatta et al 2013 | NK cells, PBL E4BP4, PBL GZMB, PBL IFN- γ, T-cells CD8+, (blood) | FACS, RT-qPCR | 2 rGBM | DC Vaccine | MRI at 6m post-vaccine start  (McDonald criteria) | Pre-vaccine start, at 22w post-vaccine start (7^th^ vaccine) | **SD at 6m (n=1):** E4BP4 increased at 7^th^ vaccine (p<0.0001) PBL IFNγ, PBL GZMB, NK cells and CD8+ T cells: no change at 7^th^ vaccine **TP at 6m (n=1):** no change all biomarkers |
| Phuphanich et al 2009* | Tc-cell CD8+ response (antigen specific) (blood) | Not specified | 15 rGBM | DC vaccine | MRI every 2m (McDonald criteria) | 56d Post-DC vaccine | **TP**: 17% (1/6) CTL responders, 56% (5/9) non-responders  **Diagnostic performance of CTL non-response for TP**  CTL non-responders: Sn: 83%, Sp:55% +LR = 1.88 –LR = 0.30 DOR = 6.27 |
| **Combination therapy/others** |  |  |  |  |  |  |  |
| Hunn et al, 2015 | Mononuclear cells IFN-γ (blood) | IFN-γ ELISpot assay | 9 rGBM | Surgery + DC vaccine + TMZ | MRI pre-1^st^ vaccine, post-3^rd^ vaccine, then every 8w (WHO criteria) | Post 1^st^ and 3^rd^ DC vaccine, then every 4w | **At time of SD**: 5/7 timepoints IFN-gamma response to at least one stimulant **At time of PR**: 2/2 timepoints showed IFN gamma response to at least one stimulant; **At time of TP**: 8/14 timepoints showed lack of IFN gamma response to any stimulant |
| Pellegatta et al, 2018 | NK cells:  %ABCC3+,  %CD56bright, %CD56dim, %GZMB+, %IFN-γ+, counts  PBL ABCC3, IFN-γ expression  T-cells CD4+:  %ABCC3+, %IFN-γ+, counts  T-cells CD8+:  %ABCC3+, %GZMB+, %IFN-γ+, counts | FACS, IFN-γ ELISpot, qRT-PCR | 24 GBM | RTx/TMZ + DC vaccine/TMZ +/- 3^rd^ line CTx/RTx/CRTx/or Bev | MRI at 9w then every 2m or earlier if suspected TP (RANO criteria) | Pre-vaccine; at 9w, 11w, 13w, 15w, 19w, 23w and 31w (1^st^-7^th^ vaccine); then every 2m until progression | **TR/SD at 12m vs TP at 12m:**  Biomarkers increased in TR/SD cases but not within progression time period for TP cases (p at least <0.01):  NK cells % ABCC3+: increased at 9, 11, 15 and 23w vs pre-vaccine  NK cells % CD56dim: increased at 11, 13, 19 and 23w vs 9w  NK cells % GZMB+: increased at 11, 19 & 23w vs pre-vaccine  NK cells % IFN-γ+: increased at 9, 13, 15, 19, 23, 31 & 37w vs pre-vaccine  PBL ABCC3 expression: increased at 9, 11, 13, 15, 19, 23, 31 and 37w vs pre-vaccine  PBL IFN- γ: increased at 13, 15 and 31w vs pre-vaccine  T-cells CD4+ % ABCC3+: increased at 9, 11 and 19w vs pre-vaccine  T-cells CD4+ %IFN- γ+: increased at 15, 19, 23, 31 and 37w vs pre-vaccine  T-cells CD8+ Counts: increased at 11 & 13w vs 9w  Other biomarkers not demonstrating differential expression between TR/SD and TP |
| Ruhle et al, 2017 | B cells, dendritic cells, eosinophils, monocytes, neutrophils, NK cells, T cell CD4+:CD8+ ratio, TLC, WCC (blood) | FACS | 1 GBM | Surgery + TMZ/RTx + Bev/Irinotecan | MRI + histopathology (Criteria NOS) | Multiple postoperative timepoints | **At TP:** Rise in monoctyes and neutrophils; Reversal of CD4:CD8 T cell ratio to CD4 predominant; other cell types not clearly changed |
| Sakai et al, 2017 | Tc-cell CD8+ T-cells CD8+ WT1 specific (blood) | FACS WT1 tetramer analysis | 2 AA (WHO III) | RTx/TMZ + WT1 DC vaccine + IFN-β (n=1 patient); RTx + nitrosourea + IFN-β + WT1 DC vaccine (n=1 patient) | MRI post-1^st^ & 2^nd^ course vaccine (Criteria NOS) | Baseline, post-1^st^ & 2^nd^ course vaccine | **SD:** corresponded with increase in % CD8+T-cells that were WT1-tetramer+ post 1^st^ course (n=1) and post 1^st^ & 2^nd^ course (n=1) |
| Shah et al 2007 | Neutrophils, Platelets, WCC (blood) | Not specified | 14 rHGG | Imatinib + hydroxyurea | MRI post-Rx (McDonald criteria) | During treatment – multiple timepoints | **TP vs SD/PR**: SD/PR associated with grade 3-4 haematological toxicity^iv^ (p=0.03) |

**Table A9:** +LR = positive likelihood ratio; -LR = negative likelihood ratio; AA: anaplastic astrocytoma; ABCC3: ATP Binding Cassette Subfamily C Member 3; Bev: Bevacizumab; CD: cluster of differentiation; CRTx: chemoradiotherapy; CTx: chemotherapy; d: days; DC: dendritic cell; DOR = diagnostic odds ratio; E4BP4: E4 promoter–binding protein 4; ELISpot: Enzyme linked immunosorbent spot; FACS: fluorescence activated cell sorting; FoxP3: forkhead box P3; GBM: glioblastoma, GZMB: granzyme B; HC: healthy control; HGG: high grade glioma; HLA-DR: Human Leukocyte Antigen- DR isotype; IFN: Interferon; m: months; Mo-MDSC: monocyte myeloid-derived suppressor cell; MPV: mean platelet volume; MDT: multidisciplinary team; NK: natural killer; NKG2D: natural killer group 2D; NLR: neutrophil to lymphocyte ratio; PBL: peripheral blood lymphocytes**;** PDW: platelet distribution width; PR: partial response; pre-op: preoperative; PsP: pseudoprogression; r: recurrent; RANO: response assessment in neuro-oncology; RT-qPCR: quantitative reverse transcription polymerase chain reaction; RTx: radiotherapy; SD; stable disease; Sn = sensitivity; SP = specificity; Tc: Cytotoxic T-cell; TLC: total lymphocyte count TMZ: temozolomide; TP: tumour progression, TR: tumour recurrence; Treg: T-regulatory; VNN2: Vascular non-inflammatory molecule 2; w: weeks; WCC: white cell count; WHO: world health organisation; WHO I, II, II or IV: WHO grade I, II, III, or IV glioma; WT1: Wilms' tumour 1.

i: NLR decrease and NLR increase were, respectively, an increase or decrease by 1 standard deviation, in NLR value closest to TP or treatment completion compared with the pre-CRTx value. ii: DVI: DR-Vanin Index, the ratio of percentage of HLA-DR- cells among CD14+ monocytes to percentage of VNN2+ cells among CD14+ monocytes

iii: only 4 patients were tested via tetramer assay.

iv: haematological toxicity grade was defined based on NCI Common Terminology Criteria for Adverse Events

*= abstract

**Table A10. Other protein biomarkers**

| **Study** | **Biomarker** | **Assay technique** | **Sample size Histology** | **Treatment** | **Reference Standard** | **Sampling points** | **Results** |
| --- | --- | --- | --- | --- | --- | --- | --- |
| **Standard treatment** |  |  |  |  |  |  |  |
| Iwamoto et al 2011 | YKL-40 (serum) | ELISA | 41 LGG, 105 AG, 197 GBM | CRTx | MRI at enrolment, every 2-3m  (RECIST & Macdonald criteria) | At enrolment; every 2-3m (within 30 days of matched MRI) | **CR vs PR/SD/TP:** YKL-40 levels lower in anaplastic glioma (p=0.0008), GBM (p=0.0006). No difference in LGG.  **Diagnostic performance of YKL-40 level in CR vs PR/SD/TP:**  Anaplastic glioma: AUC = 0.65 [95% CI 0.60-0.69], GBM AUC =0.65 [95% CI 0.61-0.70] LGG AUC = 0.56 [95% CI 0.50 – 0.61] |
| Wu et al 2019 | MGMT autoantibodies (serum) | Peptide micro-array | 63 glioma NOS (cross-sectional cohort) /10 WHO II-IV (prospective cases) | Surgery, Rx NOS | PET-MRI and MRS at TP (Criteria NOS) | 30d post-op, at TP | **TP vs 30d post-op:** MGMT-02, MGMT-07 and MGMT-10 antibodies coverage^i^ elevated (p: NR); no increase for MGMT-04 and MGMT-18  5/5 initially seropositive patients showed elevated MGMT-02 antibodies at TP  0/5 initially seronegative cases did not develop seropositive response at TP |
| Yovino et al 2012* | GFAP, neurogranin, ICAM-5, BDNF, B-syn-nuclein (serum) | ELISA | 1 rGBM  2 PsP | TMZ + RTx ± repeat surgery | Histopathology | last week CRTx; 1m, 6m, 1y post-CRTx | **PsP vs TP:** no trend difference for all biomarker levels at all timepoints |
| Anti-angiogenic therapy |  | | | | | | |
| Pace et al, 2018 | Hb (blood) | FACS | 49 rHGG | BEV | MRI at 3m (RANO criteria) | pre-2^nd^ cycle Rx, pre-3^rd^ cycle Rx | **PR vs SD vs TP:** no differences between response outcomes at all timepoints (p>0.05) |
| **Combination therapy/others** |  |  |  |  |  |  |  |
| Porter et al 2018/19* | NfL, GFAP, Tau protein | ELISA | 15 glioma NOS | Not specified | MRI at enrolment (RECIST criteria) | At enrolment | **TP vs SD:** NfL concentration elevated (p=0.0307); GFAP concentration elevated (0.0348); Tau protein concentration not different |
| Sampath et al 2004 | Recoverin (serum) | ELISA | 7 GBM | Not specified | Not specified | At enrolment | **TP vs CR:** Expression trended higher but not significant |
| Shah et al 2007 | Haemoglobin (blood) | Not specified | 14rHGG | Imatinib + hydroxyurea | MRI post-Rx (McDonald criteria) | During treatment: multiple timepoints | **TP vs SD/PR**: disease control associated with grade 3-4 haematological toxicity (p=0.03)^ii^ |
| Vietheer et al, 2017 | GFAP (serum) | ELISA | 26 GBM | RTx + TMZ ± Lomustine or Bev | MRI at TP (RANO criteria) | post-RTx, 20w, 26w, 32w, 42w, 52w or TP/death | **TP:** 1/18 TP cases with increase in GFAP 40 weeks post TP. GFAP undetectable in all other patients. |

**Table A10 legend:** AG: anaplastic glioma; AUC: area under the curve; Bev: bevacizumab; BDNF: brain-derived neurotrophic factor; CE: contrast-enhanced; CI: confidence interval; CR: complete response; CRTx: chemoradiation, d: days; ELISA: enzyme-linked immunosorbent assay, FLAIR: fluid attenuated inversion recovery; GBM: glioblastoma; GFAP: glial fibrillary acidic protein; Hb: haemoglobin; HGG: high grade glioma; ICAM-5: Intercellular adhesion molecule 5; LGG: low grade glioma, m: months; MGMT: O‐6‐methylguanine‐DNA methyltransferase; MRI: Magnetic resonance imaging; MRS: magnetic resonance spectroscopy; NfL: neurofilament light chain; NOS: not otherwise specified; NR: not reported; PET: positron emission tomography; pre-op: preoperative; post-op: postoperative; PR: partial response; PsP: pseudoprogression; RANO: response assessment in neuro-oncology; RECIST: response evaluation criteria in solid tumours; RTx: radiotherapy; TMZ: temozolomide, SD: stable disease; TP: tumour progression; WHO I, II, II or IV: world health organisation grade I, II, III, or IV glioma; y: years; YKL-40: chitinase 3-like 1 glycoprotein

i: Coverage: ratio of the seropositive serum numbers to the whole set numbers

ii: haematological toxicity grade was defined based on NCI Common Terminology Criteria for Adverse Events

* = abstract
