# Supplementary material for: Blood Biomarkers of Glioma in Response Assessment Including Pseudoprogression and Other Treatment Effects: A Systematic Review
Source: Front Oncol. 2020 Aug 14;10:1191. doi: 10.3389/fonc.2020.01191 (PMC7456864; doi:10.3389/fonc.2020.01191)
Supplement: Additional File 3 — Results of quality assessment for each individual study. [file Data_Sheet_3.docx]

**Table A11- QUADAS-2 findings for individual studies**

|  | DOMAIN | | | |
| --- | --- | --- | --- | --- |
| STUDY | Patient selection | Index Test | Reference standard | Flow and timing |
| Extracellular vesicle related biomarkers |  |  |  |  |
| Osti et al 2018 | H | H | L | L |
| Sartori et al 2013 | L | U | H | L |
| Galbo et al 2018 | U | U | U | H |
| Koch et al 2014 | U | H | U | L |
| Shao et al 2012 | U | L | L | U |
| Circulating nucleic acids |  |  |  |  |
| Cordova et al 2019* | NP | NP | NP | NP |
| Ilhan-Mutlu et al 2012 | H | L | H | H |
| Lan et al 2018 | U | H | U | U |
| Santagelo et al 2018 | H | U | U | H |
| Shao et al 2015 | H | U | L | L |
| Siegal et al 2016 | U | U | U | L |
| Swellam et al 2019 | U | U | H | U |
| Yang et al 2013 | U | H | U | L |
| Yue et al 2016 | U | H | U | U |
| Faiq et al 2015* | NP | NP | NP | NP |
| Faria et al 2018 | H | H | H | U |
| Noroxe et al 2019 | L | H | U | H |
| Circulating tumour cells |  |  |  |  |
| Gao et al 2016 | H | H | H | L |
| Macarthur et al 2014 | H | H | H | L |
| Stragliotto et al, 2016* | NP | NP | NP | NP |
| Sullivan et al 2014 | U | H | H | U |
| Angiogenesis and inflammation related proteins |  |  |  |  |
| Iwamoto et al 2011 | U | U | H | H |
| Kesari et al 2008 | L | U | H | H |
| Lin et al 2009 | U | U | H | L |
| Lin et al 2013 | U | U | H | L |
| Pellegatta et al 2013 | H | U | H | L |
| Batchelor et al 2010 | L | U | H | L |
| de Groot et al 2011 | U | L | H | L |
| Labussière et al 2016 | U | U | L | L |
| Pace et al 2018 | U | L | U | H |
| Chinnaiyan et al 2012 | U | U | U | H |
| Eoli et al 2012* | L | L | L | H |
| Gomes et al 2011* | L | U | U | U |
| Lee et al 2015 | L | U | H | H |
| Shehan et al 2019 * | U | U | L | L |
| Tabouret et al 2015 | L | U | H | L |
| Circulating angiogenesis related cells |  |  |  |  |
| Cuppini et al 2013 | L | U | L | L |
| Greenfield et al 2009 | H | U | H | U |
| Batchelor et al 2007 | L | U | H | L |
| Galanis et al 2013 | U | U | U | L |
| Alterations to Immune related and other cell lines |  |  |  |  |
| Hassan et al, 2017* | NP | NP | NP | NP |
| Huang et al 2019 | U | U | U | U |
| Parsa et al 2010 | NP | NP | NP | NP |
| Soler et al 2017 | L | H | L | L |
| Sturla et al 2013* | NP | NP | NP | NP |
| Okada et al 2011 | U | U | H | L |
| Phuphanich et al 2009 | NP | NP | NP | NP |
| Hunn et al 2015 | U | H | H | L |
| Pellegatta et al 2018 | U | U | U | L |
| Ruhle et al 2017 | H | L | U | L |
| Sakai et al 2017 | H | U | U | U |
| Shah et al 2007 | L | U | H | L |
| Other circulating proteins |  |  |  |  |
| Iwamoto et al 2011 | U | L | H | H |
| Wu et al 2019 | U | H | U | U |
| Yovino et al 2013 | NP | NP | NP | NP |
| Porter et al 2018/19* | U | L | H | L |
| Sampath et al 2004 | L | U | U | U |
| Vietheer et al 2017 | U | H | U | L |
| Legend: *= abstract; H = high; L=low; U = unclear; NP = quality assessment not performed | | | | |
